# Ezetimibe Engineered L14‐8 Suppresses Advanced Prostate Cancer by Activating PLK1/TP53‐*SAT1*‐Induced Ferroptosis

**DOI:** 10.1002/advs.202504192

**Published:** 2025-06-19

**Authors:** Yu Zhang, Xiao‐wen Song, Na Zhang, Xue‐hui Li, Fan‐chen Wu, Yu‐ang Wei, Dong‐liang Xu, Ling‐fan Xu, Fu‐wen Yuan

**Affiliations:** ^1^ Shanghai Frontiers Science Center for Chinese Medicine Chemical Biology Institute of Interdisciplinary Integrative Medicine Research Shanghai University of Traditional Chinese Medicine Shanghai 201203 China; ^2^ The Center for Cancer Research School of Integrative Medicine Shanghai University of Traditional Chinese Medicine Shanghai 201203 China; ^3^ School of Chemistry and Chemical Engineering Henan Normal University Xinxiang Henan 453007 China; ^4^ Department of Laboratory Medicine Shanghai East Hospital Tongji University School of Medicine Shanghai 200123 China; ^5^ Shanghai TCM‐Integrated Hospital Shanghai University of Traditional Chinese Medicine Shanghai 200082 China; ^6^ Department of Urology Shuang Hospital Shanghai University of Traditional Chinese Medicine Shanghai 201203 China; ^7^ Department of Urology The First Affiliated Hospital of Anhui Medical University Hefei 230001 China

**Keywords:** drug design and optimization, ferroptosis, prostate cancer, SAT1, TP53

## Abstract

Androgen receptor signaling inhibitors (ARSIs) have demonstrated a survival benefit in metastatic prostate cancer. However, patients taking these agents inevitably acquire resistance and even develop neuroendocrine prostate cancer (NEPC), in which stage the AR signaling is inactive, and therapies are limited for these lethal cases. Therefore, developing novel treatments independent of the AR signaling pathway is urgently needed. Here it is reported that L14‐8, a small molecule is derived and optimized from ezetimibe, a marketed drug primarily used for intestinal cholesterol and phytosterol absorption, significantly suppresses cell growth in advanced prostate cancer by inducing ferroptosis. Mechanistically, L14‐8 binds to and promotes the ubiquitin‐mediated PLK1 degradation, resulting in an increase of downstream TP53 protein phosphorylation, which is further enriched at the promoter of *SAT1*, a well‐established ferroptosis inducer, and boosting *SAT1* transcription thus triggers ferroptosis‐mediated cancer cell death. Importantly, in vivo studies further demonstrate a potent anti‐tumor efficacy of L14‐8 without obvious toxicity. Overall, this study develops a novel small molecular engineered from ezetimibe for treating lethal prostate cancer in an AR‐independent manner and provides mechanistic insights into its action by triggering PLK1‐TP53‐*SAT1* axis‐mediated ferroptosis in lethal PCa models independent of the AR signaling pathway.

## Graphical abstract

L14‐8, a small molecule derived from the marketed drug ezetimibe, potently inhibits advanced prostate cancer independent of androgen receptor signaling. L14‐8 binds PLK1, triggering its ubiquitin‐mediated degradation. This activates TP53‐dependent transcription of SAT1, inducing ferroptosis and suppressing tumor growth in prostate cancer patient‐derived organoids (PDOs) and in vivo with minimal toxicity.

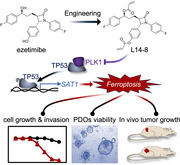

## 1 Introduction

Prostate cancer (PCa) is one of the most prevalent malignancies in men worldwide^[^ ^1^ ^]^ Given the critical role of the androgen receptor (AR) signaling pathway in tumorigenesis, surgical or medical androgen deprivation therapy (ADT) is the primary treatment for prostate cancer.^[^ ^2^ ^]^ However, patients eventually progress to castration‐resistant prostate cancer (CRPC), which contributes to the majority of prostate cancer‐related deaths. As the AR signaling pathway is continually activated in a significant number of CPPC patients due to the production of androgen outside of the testis after ADT treatment, as well as the AR amplification or overexpression within tumors,^[^ ^3^, ^4^ ^]^ recently developed AR signaling inhibitors (ARSIs), such as enzalutamide, apalutamide, darolutamide, and abiraterone, were clinically used with significant clinical efficacy in CRPC. Unfortunately, the resistance to these agents develops through mechanisms such as AR transcriptional reprogramming, AR mutations or alternative splicing, and AR‐independent lineage plasticity.^[^ ^5^ ^]^ Notably, the incidence of neuroendocrine prostate cancer (NEPC), an even more lethal stage of prostate cancer type, has increased in the era of new ARSIs. In many cases, the AR loss coincides with the activation of neuronal features, leading to treatment‐induced NEPC (t‐NEPC).^[^ ^6^, ^7^ ^]^ Due to the inactivation of the AR signaling pathway, these patients do not respond to ARSIs and succumb shortly after progressing to NEPC. Therefore, there is an urgent need to identify novel therapeutic agents to treat CRPC and NEPC.

Our recent studies revealed that *SLC7A11*, known for its role in protecting cells from ferroptosis, was significantly upregulated by the second‐generation AR antagonists, such as enzalutamide and darolutamide.^[^ ^8^ ^]^ Bedside, Viswanathan et al. identified the inhibition of the lipid peroxidase pathway, which was activated during ferroptosis, as a common feature of therapy‐resistant cancer cells across various mesenchymal cell states, including prostate cancer.^[^ ^9^ ^]^ These findings together suggested that targeting ferroptosis might be a therapeutic strategy for preventing acquired drug resistance. SAT1 (spermidine/spermine N1‐acetyltransferase 1) is a critical modulator in regulating polyamine metabolism by acetylating spermidine and spermine,^[^ ^10^ ^]^ whose overexpression leads to significant growth suppression and mitochondrial apoptosis,^[^ ^11^ ^]^ and SAT1 ablation inhibits p53‐ and p53^3KR^‐induced ferroptosis.^[^ ^12^ ^]^ Mechanistically, *SAT1* is transcriptionally regulated by TP53 and thus promotes ferroptosis in multiple cancers, and ferroptosis inhibitor ferrostatin‐1 or ALOX15 inhibitor PD146176 abolish SAT1‐mediated ferroptosis,^[^ ^12^, ^13^ ^]^ highlighting the potential of inducing ferroptosis as a cancer therapy.

The rapid construction of a small‐molecule library with structurally diverse bioactive molecules and drug analogs can significantly increase the chances of identifying potential lead compounds and offer an opportunity to discover new anti‐cancer drug candidates.^[^ ^14^, ^15^ ^]^ Drawing on our expertise in drug synthesis through emerging techniques such as photochemical synthesis and late‐stage modifications,^[^ ^16^ ^]^ we efficiently developed a novel small‐molecule library containing over 450 molecules and various drug analogs via photochemical synthesis. High‐throughput screening for anti‐tumor activity in different prostate cancer cell models with either AR expression or inactivation, which identifies six potential anti‐tumor molecules among which, subsequent cytotoxicity testing revealed that an analog, L14, derived from ezetimibe as a marketed drug previously used for intestinal cholesterol and phytosterol absorption, exhibited the best anti‐tumor activity and in vitro specificity among the six molecules. Notably, this analog demonstrated superior activity compared to the original drug ezetimibe. Inspired by these findings, we further optimized the structure of L14 through the group substitution, resulting in the analog L14‐8, which demonstrated the enhanced anti‐tumor activity by targeting and activating the PLK1/TP53‐*SAT1*‐induced ferroptosis pathway.

## 2 Results

### 2.1 Rapid Construction of Small‐Molecule Library and High‐Throughput Screening for Anti‐Tumor Activity

**Figure 1:**
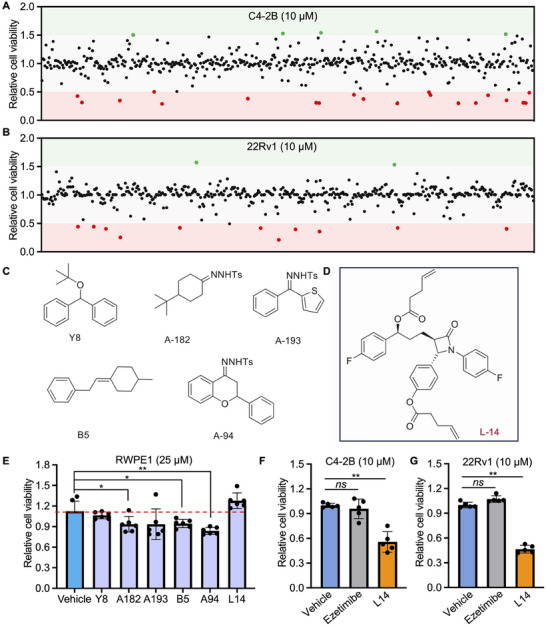
Screening of anti‐prostate cancer agents in vitro with small‐molecule library. A,B) Prostate cancer cells in C4‐2B and 22Rv1 cells were plated in 96 well plates and indicated agents were added at the concentration of 10 µm and incubated for 48 h, the cell viability was determined with a CCK‐8 kit. Marked in the red star indicates the cell growth inhibition effect of the indicated agent is consistent in both C4‐2B and 22Rv1. *n* = 3. (C,D) The structures of six agents showed tumor cell growth inhibition effects in both C4‐2B and 22Rv1. E–G) The impact of 25 µm of Y8, A182, A193, B5, A94, and L14 on normal prostate RWPE1 cell (E) and the impact of 10 µm of ezetimibe on C4‐2B and 22Rv1 cell viability determined by the CCK‐8 kit. ^**^, *p* < 0.01, ^*^ *p* < 0.05.

Prostate cancer therapy necessitates the development of new anti‐tumor drugs owing to the limitations of existing treatments, such as ARSIs. By leveraging our expertise in drug synthesis and optimization with emerging techniques,^[^ ^16^, ^17^ ^]^ we generated a small‐molecule library comprising 461 compounds. This library included seven categories with distinct core structures: hydrazones, amines, thiols, alkenes, pyrazolines, etc. For instance, the alkenes that were important pharmacophores found in many bioactive molecules were included in this collection (**Table** **1**). To evaluate the anti‐tumor potential of these compounds, we assessed their effects on cell viability in CRPC cells including C4‐2B and cells. The results indicated that Y8, A182, A94, B5, A193, and L14 significantly inhibited tumor growth in both cell lines (**Figure** **1**A–D). Further testing on the normal prostate cell line RWPE1 revealed that L14, an analog derived from ezetimibe, had the least impact on cell growth among the six compounds, compared to the vehicle (Figure 1E). Moreover, the original drug ezetimibe did not significantly affect tumor cell growth at the same dose (Figure 1F,G). These findings collectively demonstrated the anti‐tumor potential and specificity of L14 in prostate cancer.

**Table 1:** Rapid construction of small‐molecule library.

Categories	Numbers	Core structure	Representative drugs
Hydrazones	203	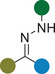	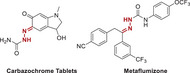
Olefins	33	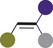	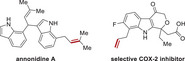
Pyrazoles, Isoxazoles	30	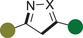	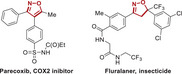
Cyclopropanes	44	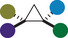	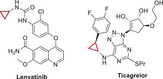
Ethers/thioethers	73	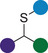	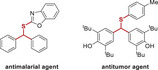
1,1,‐diaryl compounds	31	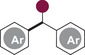	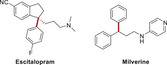
Amines	47	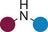	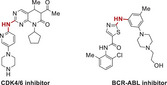

### 2.2 Structure Optimization of L14 and Structure‐Activity Relationship (SAR) Exploration

**Figure 2:**
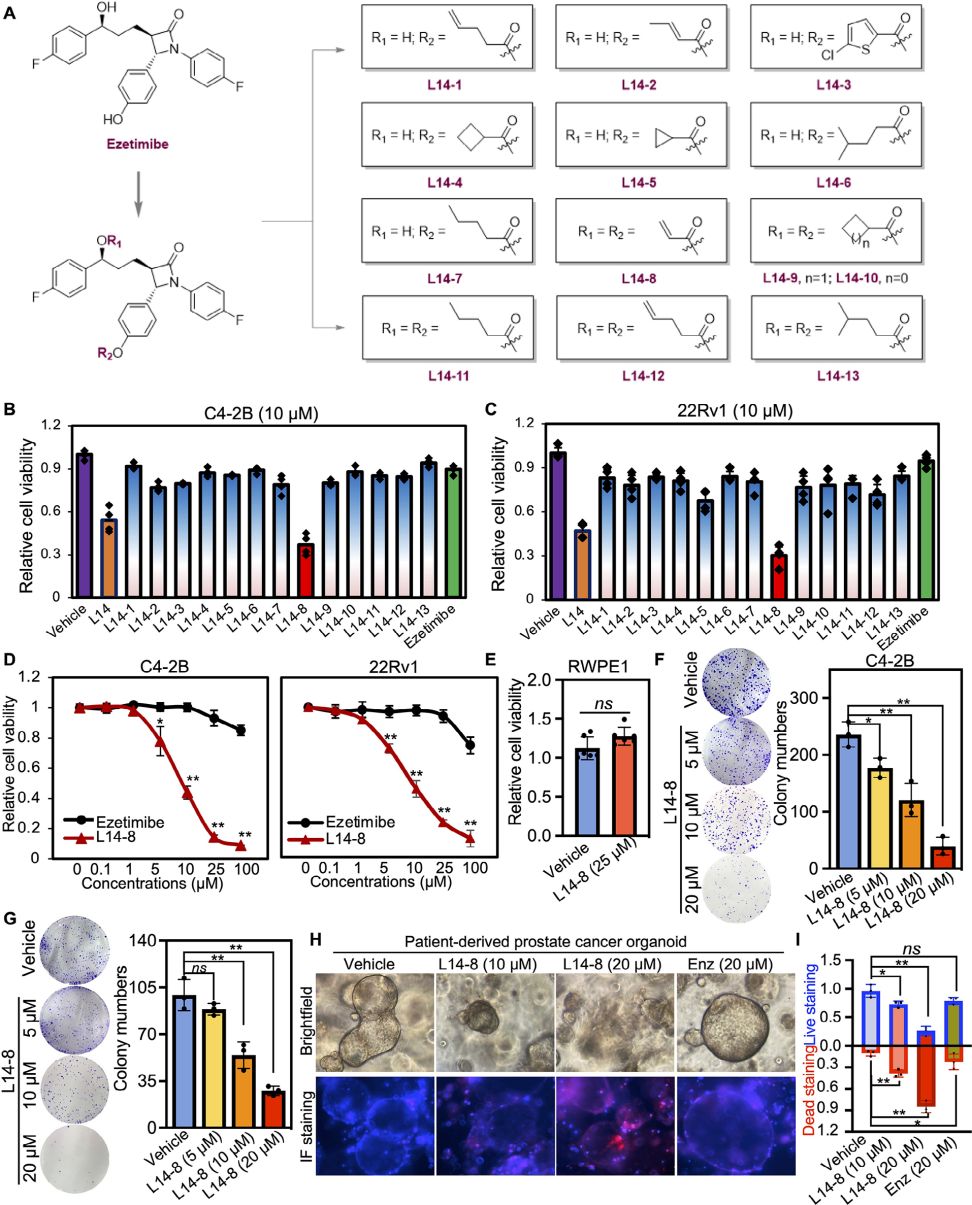
Structural optimization of Ezetimibe‐based anti‐tumor agents. A) Structures of optimized agents derived from L14 and Ezetimibe. B,C) Prostate cancer cells C4‐2B (B) and 22Rv1 (C) were plated in 96 well plates and indicated agents were added at the concentration of 10 µm and incubated for 48 h, the cell viability was determined with a CCK‐8 kit. D,E) The impact of different dosages of L14‐8 in CRPC cells C4‐2B and 22Rv1 (E) and normal prostate RWPE1 cells (E) was determined by the CCK‐8 kit. F,G) Colony formation assays to determine the impact of different doses of L14‐8 on the survival ability of C4‐2B (F) and 22Rv1 (G). H,I) Patient‐derived prostate cancer organoids were treated with indicated agents, and the morphology and viability were detected by brightfield imaging and staining with PI (red, dead) and Hoechst (blue, alive) fluorescence dye, respectively. The relative organoid viability was statistically analyzed as shown in the right panel (I), *n* = 3. ns, not significant, ^**^, *p* < 0.01.

To further improve the tumor inhibition efficacy of L14 in prostate cancer, various analogs of ezetimibe were successfully designed and synthesized (**Figure** **2**A). Their anti‐tumor efficacy was evaluated in C4‐2B and 22Rv1 cells (Figure 2B,C). Initially, L14 demonstrated superior anti‐tumor activity compared to the original drug. To further improve the activity, seven monosubstituted derivatives, L14‐1–L14‐7, including different α, β‐unsaturated and saturated esters, were synthesized successfully. However, those ezetimibe derivatives showed limited activity compared with L14. Subsequently, the disubstituted ezetimibe derivatives L14‐8–L14‐12 incorporating disubstituted α, β‐unsaturated and saturated esters were synthesized via esterification of the aliphatic hydroxyl group. The impact of these derivatives on the viability of C4‐2B and 22Rv1 cells indicated that L14‐8, bearing two additional Michael acceptors, exhibited the most significant cell growth inhibition (Figure 2A–C). The IC_50_ of L14‐8 was less than 10 µm in both C4‐2B and 22Rv1 cells (Figure 2D). Notably, even at a high dose of 25 µm, which resulted in over 80% cell death in both C4‐2B and 22Rv1 cells, L14‐8 did not significantly affect normal prostate cell growth (Figure 2E), highlighting its promising specificity in prostate cancer treatment. Further colony formation assays demonstrated the strong inhibition of tumor survival (Figure 2G–J). Moreover, the treatment of L14‐8 in prostate cancer organoids derived from patients who underwent ADT demonstrated a significant induction of cell death compared to the AR antagonist enzalutamide (Figure 2H,I; Figure S1, Supporting Information). These results presented the potential anti‐tumor effect of L14‐8. Notably, combining L14‐8 with low dosages of AR antagonists, such as enzalutamide and darolutamide, significantly enhanced their anti‐tumor activity in the AR‐positive prostate cancer cell models (Figure S2, Supporting Information). This suggested that combining L14‐8 with low doses of AR antagonists could potentially address the AR antagonist‐induced drug resistance, as current clinical dosages of AR antagonists, such as enzalutamide (25 µm) and darolutamide (15 µm) rapidly induced the AR transcription reprogramming and drug resistance compared with the dosages we used in this study as we reported previously.^[^ ^8^ ^]^

### 2.3 L14‐8 Induced Ferroptosis by Transcriptionally Activating *SAT1* expression

**Figure 3:**
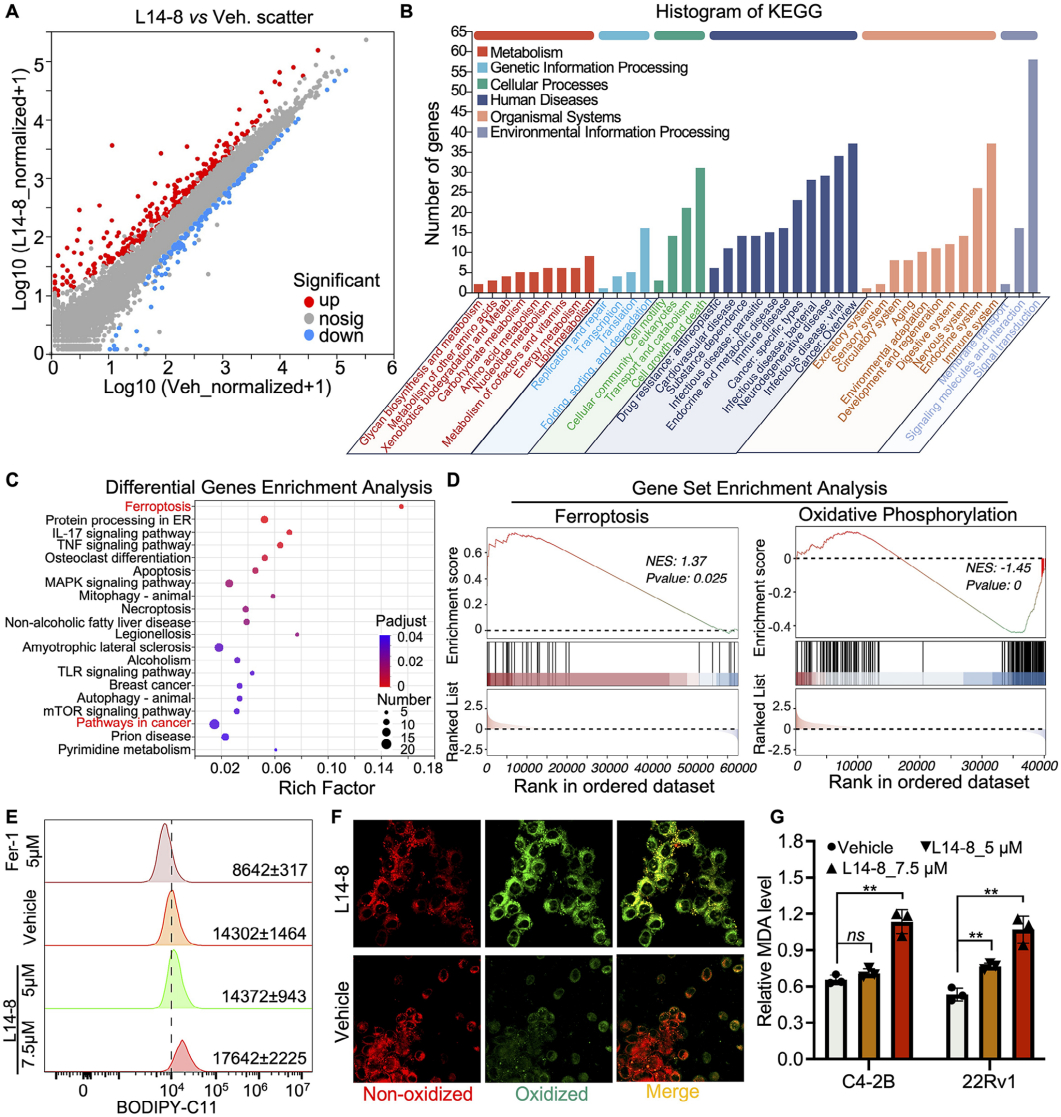
Transcriptome analysis reveals that L14‐8 induced ferroptosis in prostate cancer. A) RNA sequencing to character the differential expression genes (DEGs) after L14‐8 treatment in the prostate cancer cell model. B) Histograms of the gene count that fall into different KEGG pathways. C,D) Differential gene enrichment analysis and GSEA analysis of DEGs after the L14‐8 treatment highlighted the significance of ferroptosis. E,F) flow cytometry analysis (E) and microscope image analysis of BODIPY‐C11 after cells were treated with L14‐8. G) The MDA level after indicated cells were treated with different dosages of L14‐8 was determined with the Lipid Peroxidation MDA Assay Kit after cells were treated for 48 h. ns, not significant, ^**^, *p* < 0.01.

**Figure 4:**
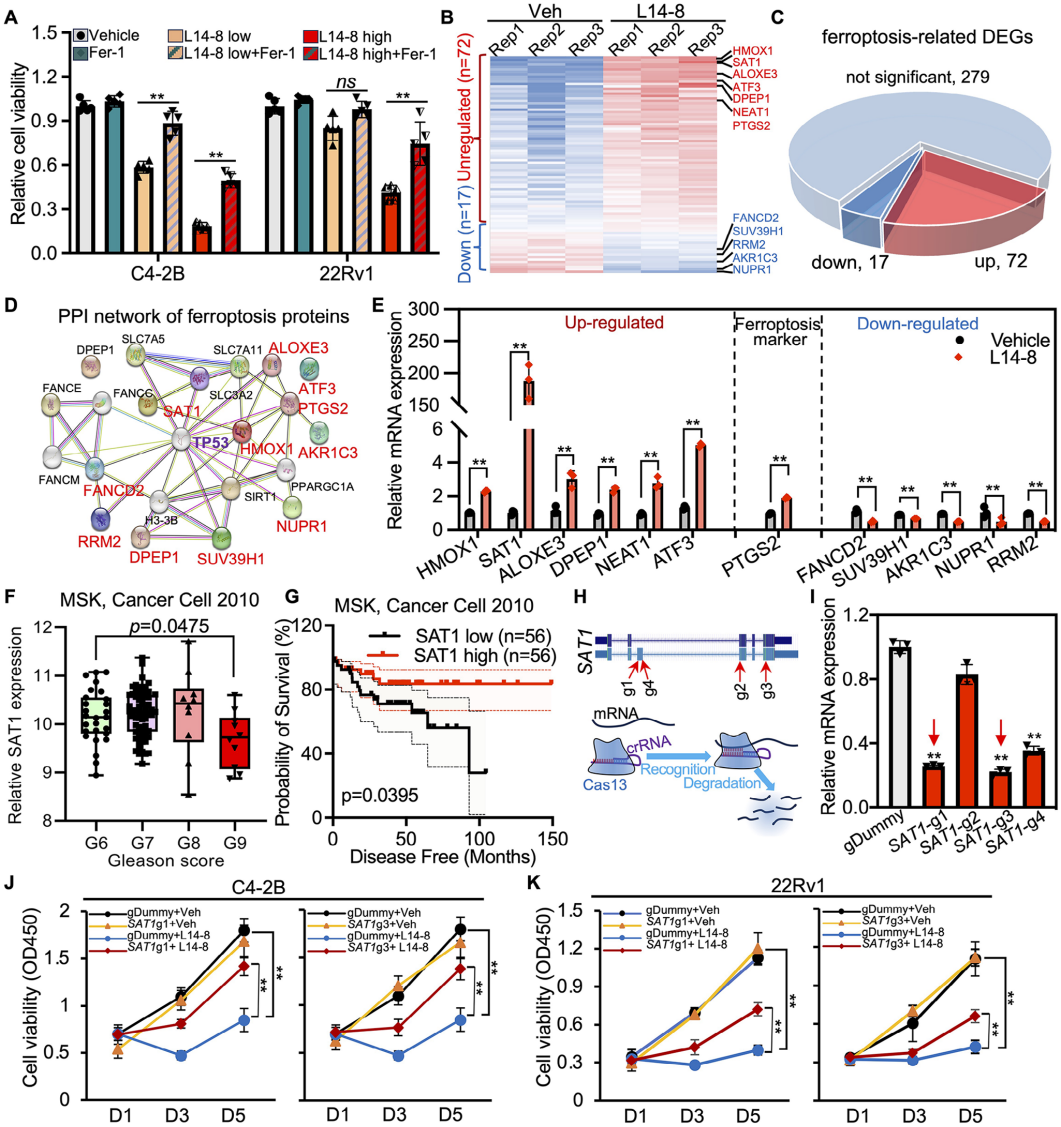
L14‐8‐induced *SAT1* transcriptional activation triggered ferroptosis. A) CCK‐8 assays were employed to determine the rescue effect of ferr‐1 on L14‐8 treatment‐induced cell growth inhibition in C4‐2B and 22Rv1 cells. B,C) RNA‐seq analysis of ferroptosis‐related gene expression analysis after L14‐8 treatment. D) Protein–protein interaction (PPI) analysis of DEGs with TP53. E) RT‐qPCR analysis of ferroptosis‐related gene expression analysis after L14‐8 treatment. F,G) The expression correlation of SAT1 with Glasson score (F) and patient survival (G) in MSK prostate cancer cohort. H,I) Gene ablation efficiency of different gRNAs corresponding to CRISPR‐Cas13 targeting *SAT1*. J,K) Cell viability was determined by CCK‐8 assays after *SAT1* was knocked down and treated with L14‐8 in C4‐2B (H) and 22Rv1 (I) cells. ^**^, *p* < 0.01.

Given the significant tumor suppression efficacy of L14‐8 in different lethal prostate cancer models, we next asked how L14‐8 is involved in tumor growth inhibition. Transcriptome analysis was then performed following L14‐8 treatment (**Figure** **3**A), and the pathways analysis of differentially expressed genes (DEGs) revealed the highest number of DEGs in the pathways of “Lipid metabolism”, “Cell growth and death”, and “Cancer: Overview” after L14‐8 treatment. Consistently, Differential Gene Enrichment Analysis (DGEA) and Gene Set Enrichment Analysis (GSEA) indicated significant enrichment in the ferroptosis pathway, suggesting that ferroptosis was involved in the L14‐8‐mediated tumor growth suppression (Figure 3B–D; Table S1, Supporting Information). Further analysis using C11‐BODIPY(581/591), a fluorescent probe for lipid peroxidation and antioxidant efficacy indicative of ferroptosis in living cells,^[^ ^18^, ^19^, ^20^ ^]^ demonstrated the significant activation of ferroptosis following the L14‐8 treatment (Figure 3E,F; Figure S3, Supporting Information). Further lipid peroxidation (LIP) assays demonstrated a dose‐dependent induction of MDA level in both C4‐2B and 22Rv1 cells (Figure 3G). Additionally, supplemental ferroptosis inhibitor ferr‐1 significantly rescued the tumor cell growth induced by L14‐8 (**Figure** **4**A). These data collectively revealed the ferroptosis effect of L14‐8 in prostate cancer. It is worth noting that, although the apoptosis pathway was also enriched in the RNA‐seq DEGs analysis (Figure 3C), significant cellular apoptosis was observed only at higher doses of L14‐8 in prostate cancer cell models, as determined by flow cytometry (Figure S4A–D, Supporting Information), except for PC3, in which cells, a low dosage of L4‐8 significantly induced cellular apoptosis (Figure S4A–D, Supporting Information).

To further determine the genes that mediated L14‐8 induced ferroptosis, we next analyzed the expression profile of ferroptosis‐related genes following the L14‐8 treatment and observed that 72 genes were upregulated, and 17 genes were downregulated (Figure 4B,C). Notably, the protein–protein interaction (PPI) analysis indicated that a significant number of the L14‐8 upregulated genes were associated with TP53, a well‐known regulator of ferroptosis (Figure 4D). Real‐time qPCR further confirmed the impact of L14‐8 on the expression of ferroptosis‐related genes, with *SAT1* demonstrating a dramatic increase of hundreds of times greater than that in the control (Figure 4E), whose upregulation has previously been reported to promote ferroptosis.^[^ ^12^, ^21^ ^]^ Indeed, survival analysis across different prostate cancer patient cohorts revealed a negative correlation between *SAT1* expression and both prostate cancer prognosis and Gleason score (Figure 4F,G; Figure S5, Supporting Information). And knockdown of *SAT1* using the cutting‐edge CRISPR‐Cas13 technology (Figure 4H,I) effectively abolished the tumor growth inhibition triggered by L14‐8 (Figure 4J,K). Collectively, these findings demonstrate that L14‐8 induced ferroptosis by activating the *SAT1* expression.

### 2.4 TP53 is Required for L14‐8 Enabled *SAT1* Transcription

**Figure 5:**
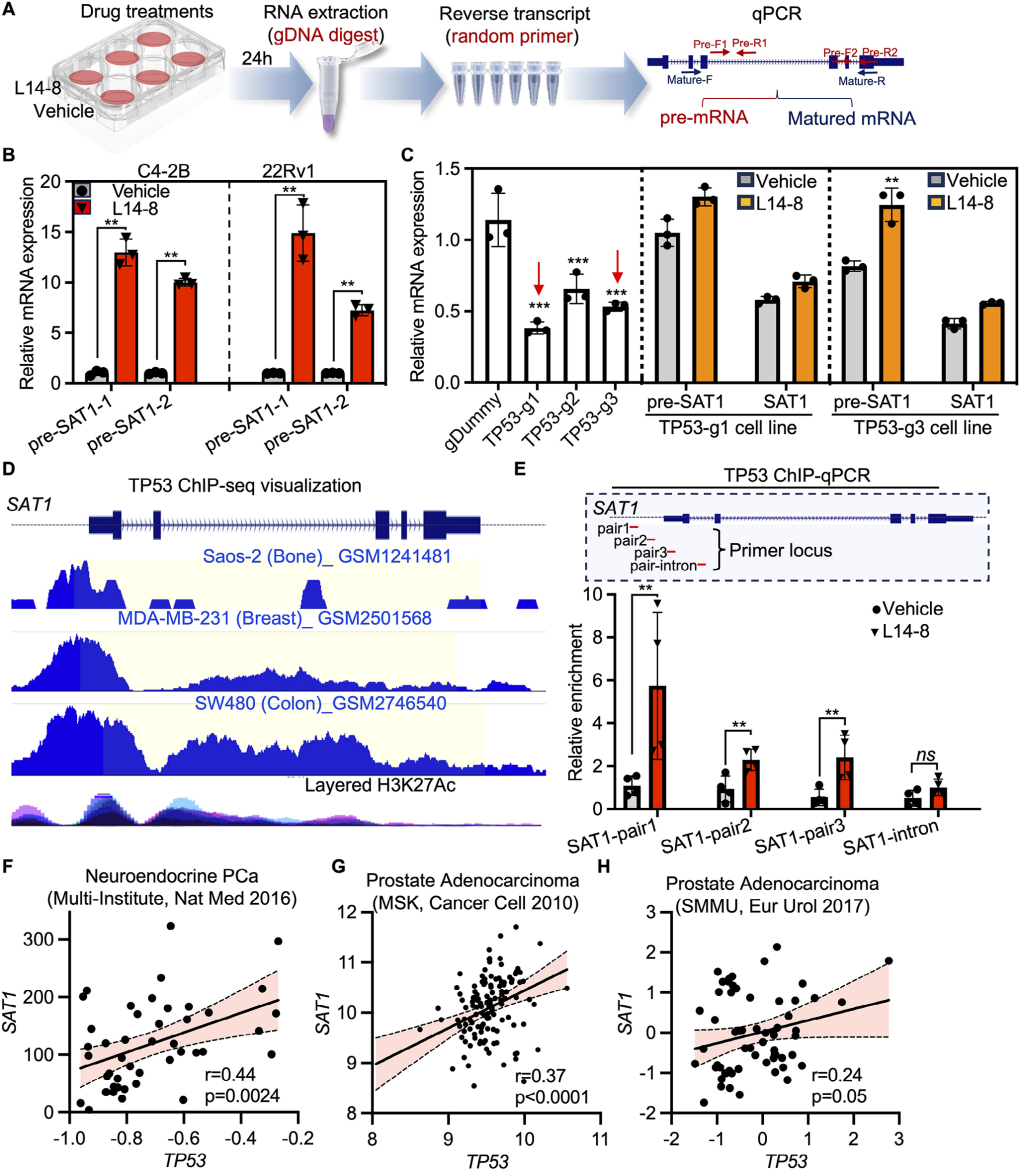
Short TP53 is indispensable for L14‐8‐induced *SAT1* transcriptional activation. A) Flow diagram to generate premature mRNA (pre‐mRNA) library for pre‐*SAT1* detection. B) RT‐qPCR to detect the relative pre‐SAT1 mRNA expression after cells were treated with L14‐8. C) The left panel indicated the knockdown efficiency of different gRNAs corresponding to CRISPR‐Cas13 targeting *TP53*, and the middle and right panels showed the expression of pre‐*SAT1* and SAT1 in *TP53* ablated cells after being treated with L14‐8. D) TP53 ChIP‐seq visualization of the enrichment of TP53 on the promoter of *SAT1* in different cancer cell models. E) ChIP qPCR to determine the enrichment of TP53 on SAT1 promoter after treated with L14‐8. The upper panel shows the location of primers used for ChIP qPCR analysis. F–H) Expression correlation analysis of TP53 and SAT1 in different prostate cancer cohorts. ns, not significant, ^***^, *p* < 0.001, ^**^, *p* < 0.01.

The mRNA level is tightly regulated by transcription and post‐transcriptional processes such as RNA maturation, modification, and degradation.^[^ ^22^, ^23^ ^]^ Here we asked in which way the *SAT1* mRNA expression is boosted by L14‐8. We collected both premature and matured mRNA after cells were treated with L14‐8, and the premature and mature *SAT1* mRNA were respectively amplified with primers targeting the intron and exons spanning introns (**Figure** **5**A). The results presented the significant upregulation of both premature and mature *SAT1* mRNA by L14‐8, indicating that L14‐8 affected the *SAT1* mRNA through transcriptional regulation (Figures 4D and 5B). Given the central role of TP53 in the L14‐8‐regulated genes (Figure 4D) and the known transcriptional regulation of *SAT1* by TP53 in triggering ferroptosis,^[^ ^24^ ^]^ we investigated whether TP53 was necessary for the L14‐8‐induced activation of *SAT1* transcription. Knockdown of *TP53* using CRISPR‐Cas13 revealed that *TP53* depletion significantly abolished the *SAT1* upregulation induced by the L14‐8 treatment (Figure 5C). ChIP‐seq analysis in various cancer cell models further indicated the significant enrichment of TP53 at the *SAT1* promoter (Figure 5D), and ChIP‐qPCR exhibited the enhanced binding of TP53 to the *SAT1* promoter, with a further increase upon the L14‐8 treatment (Figure 5E). Moreover, the *SAT1* expression was positively correlated with *TP53* in different prostate cancer cohorts (Figure 5F–H). These findings collectively demonstrated that TP53 was essential for the L14‐8‐induced *SAT1* transcriptional activation, which triggered ferroptosis.

### 2.5 L14‐8 Binds to PLK1 and Enhances PLK1 Meditated TP53 Phosphorylation and Expression

**Figure 6:**
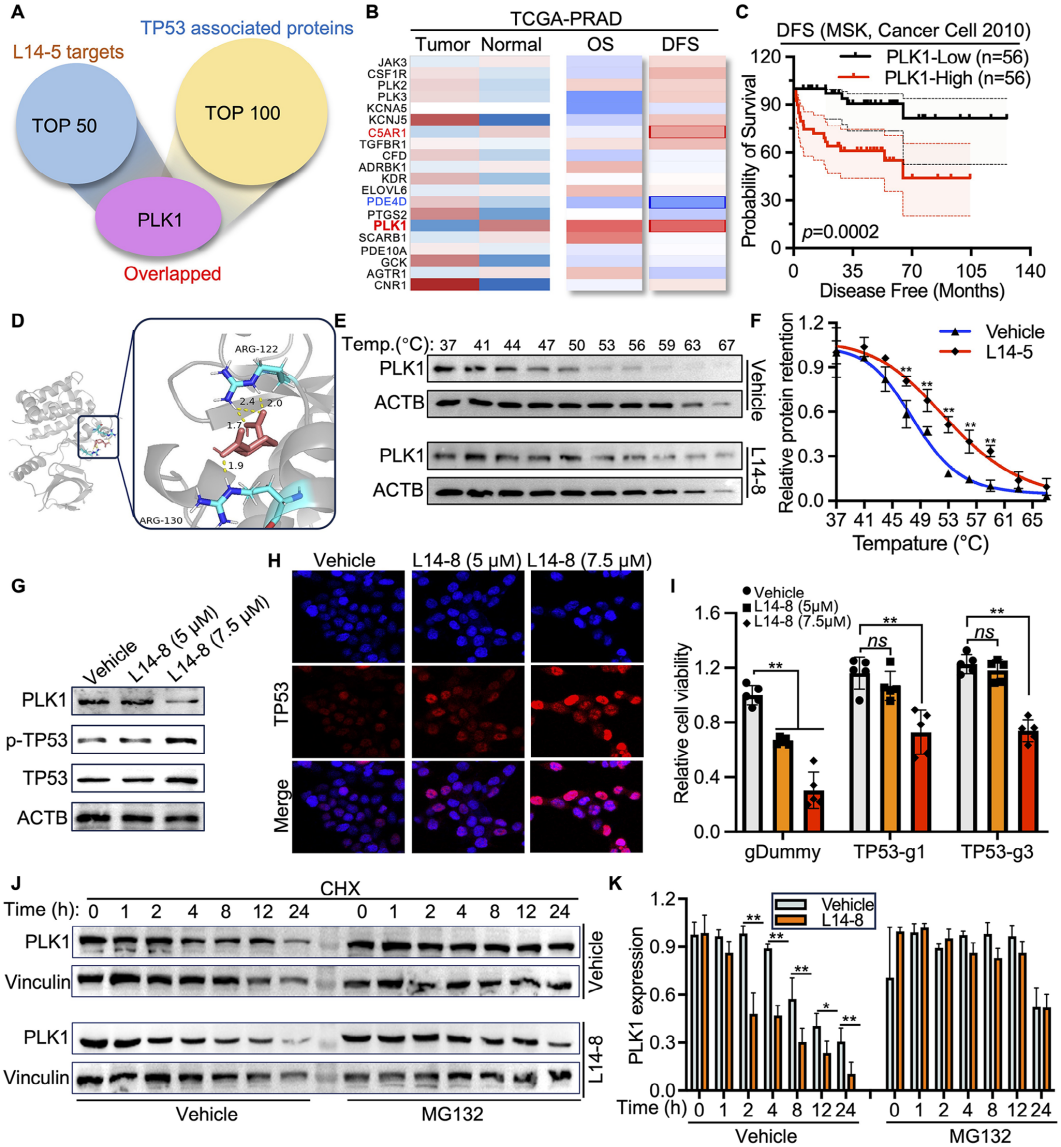
L14‐8 binds to PLK1 and promotes TP53 expression and phosphorylation. A) L14‐8 targets prediction and correlation analysis of TP53‐associated proteins. B) The relative expression of the top 20 L14‐8 predicted targets in prostate tumor versus adjacent prostate tissues and their correlation with overall survival (OS) and disease‐free survival (DFS) in the TCPG‐PRAD cohort. C) DFS analysis of PLK1 with MSK prostate cancer cohort. D) Molecular docking of PLK1 with L14‐8, the binding energy is −5 kJ mol^−1^. E,F) CETSA assay to determine the thermal stability of PLK1 after cells were treated with L14‐8. The left panel shows the representative western blot images and the right shows the statistical analysis of three independent experiments. G) Western blot to evaluate the impact of L14‐8 on the protein expression of PLK1, TP53, and the phosphorylation of TP53. H) Microscope image to visualize the protein expression and localization of TP53 after cells were treated with L14‐8. I) Cell viability was detected with CCK‐8 kit after cells transfected with TP53‐targeted CIRSPR‐Cas13 were treated with indicated dosages of L14‐8 for 72 h. J,K) C4‐2B cells were treated with Cycloheximide (CHX, 40 µm), L14‐8 (10 µm), and MG132 (40 µm) for the indicated time, cells were then collected for western blot analysis with PLK1 and vinculin antibodies. ns, not significant, ^**^, *p* < 0.01.

To investigate the direct binding targets of L14‐8 involved in the TP53‐mediated ferroptosis in prostate cancer, we retrieved the predicted targets of L14‐8 from the SwissTargetPrediction portal. The top 50 targets with the highest probability were analyzed for overlap with the TP53 interaction proteins, revealing that PLK1 was the only target intersecting with TP53 (**Figure** **6**A). Notably, PLK1 was highly expressed in the prostate tumors compared to the adjacent tissues, and its elevated expression correlated with poor overall survival (OS) and disease‐free survival (DFS) in various cohorts of prostate cancer patients (Figure 6B,C; Figure S6, Supporting Information). Molecular docking revealed a binding affinity of −5.07 kJ mol^−1^ between L14‐8 and PLK1 (Figure 6D). Additionally, the CETSA assay, a well‐established method for evaluating protein‐compound interactions,^[^ ^25^, ^26^, ^27^, ^28^ ^]^ demonstrated that the L14‐8 treatment significantly increased the thermal stability of endogenous PLK1 in prostate cancer cell models (Figure 6E,F). Given that PLK1 was known to directly bind to TP53 and inhibit its transcriptional activity by reducing its protein stability and phosphorylation, we assessed the mRNA and protein expression levels of PLK1 and TP53. The RT‐qPCR results indicated that the mRNA levels of both *PLK1* and *TP53* remained unchanged following the treatment with various doses of L14‐8 (Figure S7, Supporting Information). In contrast, the L14‐8 treatment led to an increase in the protein levels of TP53 and phosphorylated TP53 (p‐TP53) (Figure 6G,H), while the PLK1 protein levels were reduced (Figure 6G). Further protein stability assays demonstrated that L14‐8 treatment induced significant PLK1 degradation in ubiquitin‐mediated manners as supplemental with proteasome inhibitor MG132 abolished L14‐8 induced PLK1 degradation (Figure 6J,K). Additionally, TP53 knockdown with CRISPR‐Cas13 significantly desensitized cells to L14‐8 (Figure 6I). These findings suggested that L14‐8 bound to PLK1, promoting its degradation and thereby enhancing the TP53 transcriptional activity through increased TP53 protein phosphorylation and expression, and thus induced cell ferroptosis. It is worth noting that despite L14‐8 being engineered and optimized from ezetimibe, which was reported to inhibit intestinal cholesterol and phytosterol absorption by targeting NPC1L1,^[^ ^29^ ^]^ they seem to exert distinctive functions as both of their targets and regulated differently expressed genes are different (Figure S8, Supporting Information).

### 2.6 L14‐8 Suppressed Prostate Cancer Growth In Vivo Without Significant Toxicity

**Figure 7:**
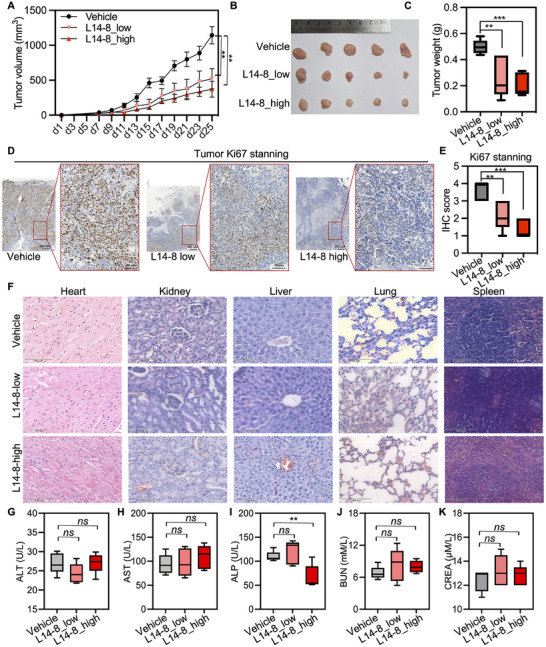
The anti‐tumor growth effect and potential toxicity of L14‐8 in C4‐2B xenografted nice model. A) Tumor volume after mice were treated with different doses of L14‐8 for indicated times. B,C) Tumor images (B) and tumor weight (C). D,E) representative images (D) and statistical analysis (e) of Ki67 staining of prostate tumors in different groups. F–H) Representative images of H&E staining of indicated major organs (F) and statistical analysis of the liver and kidney indicators including ALT, AST, ALP, BUN, and CREA G–K) after the mice were treated with different doses of L14‐8 for 25 days. *n* = 5, ns, not significant, ^***^, *p* < 0.001, ^**^, *p* < 0.01.

Encouraged by the significant tumor suppression efficacy of L14‐8 in vitro, we administered the daily doses of 10 and 20 mg kg^−1^ L14‐8, along with vehicle control, to C4‐2B xenografted mice. L14‐8 significantly suppressed tumor growth in vivo in a dose‐dependent manner (**Figure** **7**A–C). Ki67 staining, a marker of cell proliferation, revealed a marked decrease in Ki67 in the tumors treated with L14‐8 (Figure 7D,E), demonstrating its anti‐proliferative effects in vivo. Notably, the H&E staining of major organs, such as the heart, kidney, liver, lung, and spleen, exhibited no significant changes with high doses of L14‐8 compared to the vehicle (Figure 7F). The liver and kidney function indices (including ALT, AST, ALP, BUN, and CREA) were similar between the L14‐8‐treated and vehicle‐treated mice, with the exception of a decrease in ALP in the high‐dose L14‐8 group (Figure 7F–K). These results indicated that L14‐8 effectively suppressed prostate cancer growth in both in vivo and in vitro models without significant toxicity.

## 3 Discussion

ARSIs that target the AR signaling pathway have become the mainstay of treatment for various stages of prostate cancer.^[^ ^30^ ^]^ However, rapid resistance exists, and these therapies often lead to the development of NEPC, a condition characterized by an inactive AR signaling pathway that currently lacks standard treatment options.^[^ ^5^ ^]^ Additionally, ARSI therapies are associated with significant systemic disruptions, causing adverse events, such as cardiovascular events, anemia, osteoporosis, fatigue, weight gain, and hot flashes.^[^ ^31^, ^32^ ^]^ Therefore, there is an urgent need for novel anti‐tumor drugs that are not dependent on the AR signaling pathway, particularly for patients who develop resistance to ARSIs. In this study, a small‐molecule library consisting of 461 compounds was constructed using an emerging synthetic toolbox. Among these, L14 derived from the marketed drug ezetimibe was identified as a potential anti‐tumor molecule with excellent anti‐tumor activity and in vitro safety. Subsequent structural optimization of L14 led to the development of the drug candidate L14‐8, which demonstrated the superior anti‐tumor activity both in vivo and in vitro, without significant toxicity (Figures 2 and 7). These findings highlighted the substantial potential of L14‐8 in the treatment of lethal prostate cancer. Additionally, five other compounds, including Y8, A182, A94, B5, and A193, also exhibited anti‐tumor efficacy with minimal impact on normal prostate cells at higher doses. Future studies are required to evaluate their in vivo anti‐tumor efficacy and potential toxicity.

Programmed cell death (PCD) is increasingly being recognized for its role in cancer pathogenesis and treatment. Ferroptosis, a newly identified form of PCD,^[^ ^33^ ^]^ has gained attention as a potential therapeutic strategy for various diseases.^[^ ^34^ ^]^ Recent studies on prostate cancer patient‐derived organoids and cell models have indicated that prostate cancer cells are selectively sensitive to ferroptosis inducers.^[^ ^9^, ^35^ ^]^ This suggests that targeting the ferroptosis regulators can be an effective approach to induce cell death and potentially prevent acquired drug resistance in prostate cancer.^[^ ^9^ ^]^ In this study, we demonstrated that L14‐8 significantly activated the ferroptosis pathways and induced ferroptosis in CRPC and AR‐negative prostate cancer cell models, demonstrating its efficacy in suppressing cancer growth in vivo without significant toxicity (Figures 3 and 7). Notably, our findings, along with other studies, highlighted the complex role of ferroptosis in ARSI treatment, where genes such as *SLC7A11* were upregulated to inhibit ferroptosis,^[^ ^8^ ^]^ while lipid uptake and remodeling contributed to the ferroptosis hypersensitivity.^[^ ^36^ ^]^ Our study revealed that combining L14‐8 with ARSIs, such as enzalutamide and apalutamide, significantly enhanced cell death compared to either L14‐8 or ARSIs alone in CRPC cells (Figure S1, Supporting Information). In summary, these findings demonstrated the potent anti‐tumor effects of L14‐8 by inducing ferroptosis, either combined with ARSIs in the AR‐positive cancer types or alone in the AR‐negative cancers, such as NEPC.

Despite the role of L14‐8 in inducing ferroptosis through the interaction with PLK1 and the activation of the TP53‐SAT1 signaling pathway, our transcriptome analysis and phenotype assays also indicated the activation of apoptosis (Figure 3B,C; Figure S3, Supporting Information). Furthermore, the target prediction analysis identified a set of L14‐8 targets known to be established regulators of apoptosis (Table S5, Supporting Information). Considering that ablation of the TP53‐SAT1 signaling pathway did not fully abolish L14‐8‐induced cell growth inhibition, we supposed that other targets and mechanisms of L14‐8 are also involved in the anti‐tumor effects of L14‐8, for example, the induction of apoptosis as we observed in the p53‐deficient PC3 cells, while further studies are needed. Additionally, we observed a different sensitivity of C4‐2B and 22Rv1 cells to our library compounds, which we supposed was due to the genetic background differences, including the distinctive transcriptome as we discussed before,^[^ ^37^ ^]^ and the varied gene mutations among different prostate cancer cell lines, which worthy further exploration to develop new agents to targets cancer subtypes with specific genetic background.

## 4 Experimental Section

### Construction of Small‐Molecule Library

Commercial reagents were used without purification, and the reactions were conducted under an argon atmosphere (except for hydrazone synthesis) with the moisture excluded using the standard techniques for handling the air‐sensitive compounds. Unless otherwise noted, all the reactions were performed in oven‐dried glassware with magnetic stirring under an inert atmosphere of dry argon. Small molecules with various core structures were rapidly constructed, and numerous drug analogs, including those derived from ketoprofen (anti‐inflammatory), sertraline (anti‐depressive), fenofibrate (lipid‐regulating), lurasidone (anti‐psychotic), fendiline (anti‐anginal), and bifonazole (anti‐fungal), were successfully obtained through late‐stage modifications. The detailed synthesis procedures for these diverse molecules are provided in the Supporting Information.

### Structure Optimization of L14

First, it was confirmed that the L14 showed better anti‐tumor activity compared with the original drug, and showed the importance of modifying the hydroxyl groups of Ezetimibe. Therefore, seven monosubstituted L14‐1–L14‐7, including different α, β‐unsaturated esters, and saturated esters were synthesized to determine the necessity of additional groups. Subsequently, disubstituted derivatives L14‐8–L14‐12 were synthesized through subsequent esterification of the aliphatic hydroxyl group.

### Cell Viability with CCK8 Assays

The cell viability was assessed using CCK‐8 assays. 22Rv1 (RRID:CVCL_1045), PC3 (RRID:CVCL_0035), C4‐2B (RID:CVCL_4784), and RWPE1 (RID:CVCL_3791) cells were seeded in 96‐well plates at densities of 4000, 3000, 4000, and 6000 cells per well, respectively, and incubated for 24 h. The cells were then treated with the indicated agents for 72 h. Following treatment, the cells were incubated with 90 µL of medium and 10 µL of CCK‐8 reagent (GlpBio, USA) for 1 h. The absorbance at 450 nm was measured using a 96‐well plate reader (TECAN Spark, Switzerland).

### Colony Formation Assays

C4‐2B and 22Rv1 cells were seeded in the six‐well plates at densities of 1000 and 2000 cells per well, respectively. After 24 h, the cells were treated with the indicated doses of L14‐8 and incubated at 37 °C with 5% CO_2_ for an additional 12 days, with the medium supplemented with L14‐8 replaced every 2 days. The cells were fixed with methyl alcohol and stained with 1% crystal violet. The number of colonies was counted using ImageJ software (ImageJ, RRID: SCR_003070, National Institutes of Health, Bethesda, USA).

### Western Blot Assays

Western blotting was performed as previously described.^[^ ^8^ ^]^ The cells were lysed in cell lysate buffer and centrifuged at 12000 rpm at 4 °C. The protein concentration was quantified using a bicinchoninic acid (BCA) kit. The proteins were separated using 10% SDS‐PAGE and transferred to the PVDF membranes. After blocking with 5% BSA for 1 h at room temperature, the membranes were incubated overnight at 4 °C with the following primary antibodies: PLK1 (#F0393; dilution 1:1000; Selleck, Houston, USA), p‐TP53 (#F0355; dilution 1:1000; Selleck, Houston, USA), TP53 (#2524T; dilution 1:2000; Cell Signaling Technology, Boston, USA), Vinculin ((#F0110, dilution 1:2000, Selleck, Houston, USA), and ACTB (GB15001, dilution 1:2000; Servicebio, Wuhan, China). The primary antibodies were incubated on a shaker overnight at 4 °C. The membranes were then incubated with HRP‐labeled goat anti‐rabbit/mouse secondary antibodies (A0208/A0216, Beyotime, Shanghai, China), and the bands were visualized using electrochemiluminescence (ECL). For protein stability assays, MG132 (D50396s, Bioss), and Cycloheximide (DRE‐C11830000, Dr. E) were used and performed as described before.^[^ ^38^ ^]^

### RNA‐seq and Data Analysis

RNA‐seq was conducted and analyzed as previously described, with minor modifications.^[^ ^8^ ^]^ C4‐2B cells were treated with DMSO or 10 µm L14‐8 for 24 h. The total RNA was isolated using the FastPure Cell/Tissue Total RNA Isolation Kit V2 (Vazyme RC112‐01; China). The RNA quality was assessed using a Bioanalyzer 2100 (Agilent, USA), and the integrity number (RIN) for all the samples was > 9.0. The mRNA was enriched using the NEBNext Poly(A) mRNA Magnetic Isolation Module. The libraries were prepared using the NEBNext Ultra Directional RNA Library Prep Kit according to the manufacturer's instructions and sequenced using NovaSeq 6000. RNA‐seq was performed by Majorbio Co. Ltd. (China), and data analysis was conducted using the online tool of Majorbio Cloud Platform (https://cloud.majorbio.com/page/tools/).

### Real‐Time Quantitative PCR (RT‐qPCR)

RT‐qPCR was conducted as previously described, with minimal modifications.^[^ ^39^ ^]^ The cells were treated with DMSO or the indicated doses of agents for 48 h. The total RNA was extracted using the FastPure Cell/Tissue Total RNA Isolation Kit V2 (RC112‐01, Vazyme, China) and reverse‐transcribed into the first‐strand DNA using HiScript II Q RT SuperMix for qPCR (RC223‐01, Vazyme, China). The gene amplification was performed using ChamQ Universal SYBR qPCR Master Mix (Q711‐02, Vazyme, China) and analyzed in a 96‐well plate using a LightCycler 480 II machine (Roche, Switzerland). The primers used are listed in Table S2 (Supporting Information).

### Standard Chromatin Immunoprecipitation (ChIP)‐qPCR

ChIP assays were performed as previously described.^[^ ^8^ ^]^ Briefly, the cells were crosslinked with 1% formaldehyde, and chromatin was collected, sonicated, and immunoprecipitated with 2 µg of TP53 (#2524, Cell Signaling Technology, Boston, USA) or normal mouse IgG (#68860, Cell Signaling Technology, Boston, USA) antibodies at 4 °C overnight. Protein A‐Sepharose beads (#9863, Cell Signaling Technology, Boston, USA) were then added and incubated with rotation for an additional 1 h. The beads were washed sequentially with buffer I, buffer II, and buffer III, and twice with TE buffer. The chromatin complexes were eluted with the elution buffer and de‐crosslinked at 65 °C overnight. The DNA fragments were purified and analyzed by qRT‐PCR using ChamQ Universal SYBR qPCR Master Mix (Q711, Vazyme, China). The primers used for ChIP are listed in Table S3 (Supporting Information).

### Detection of Ferroptosis with BODIPY‐C11

The lipid peroxidation levels were measured using a BODIPY 581/591 C11 kit (D3861, Thermo Fisher Scientific). Following the treatment with DMSO, ferroportin‐1 (ferroptosis inhibitor), RSL3 (ferroptosis inducer, S8155; Selleck, USA), or L14‐8 for the specified durations, the cells were incubated with the BODIPY‐C11 probe at a final concentration of 5 µm at 37 °C for 30 min. For the flow cytometry analysis, the cells were digested with trypsin, collected, and centrifuged at 1000 rpm for 3 min. The cells were then resuspended in 500 µL PBS, transferred to tubes, and fluorescence was measured using a CytoFLEX‐3 cytometer (Beckman Coulter), where the dye oxidation shifted the fluorescence emission peak from 590 to 510 nm. For the confocal microscopy, the cells were imaged at 40x oil magnification with a Leica SP5 II confocal laser scanning microscope (Leica, Germany), and the image analysis was conducted using NIS‐Elements Viewer (NIKON, Japan).

### Lipid Peroxidation & Malondialdehyde (MDA) Assay

Cells were treated with vehicle and specified dosages of L14‐8 for 48 h, the cell lysates were collected, and protein was quantified using the bicinchoninic acid (BCA) kit. Subsequently, MDA concentrations were calculated with the Lipid Peroxidation MDA Assay Kit (Beyotime, S0131M) following the manufacturer's protocol. MDA concentrations were normalized to protein concentration.

### CRISPR‐Cas13 Mediated RNA Silencing

Five guide RNA (gRNA) sequences targeting CDC25B were designed for CRISPR‐Cas13 and cloned into the gRNA backbone (Addgene, 109053). The gRNA plasmids, along with CRISPR‐Cas13 plasmids (SP‐2833, CRICS, China), were transfected into cells using the ExFect Transfection Reagent (T101‐02, Vazyme, China). After transfection, RNA was extracted from the cells for RT‐qPCR to assess the deletion efficiency of the gRNAs, and two gRNAs were selected for further assays. All the gRNA sequences are listed in Table S4 (Supporting Information).

### Patient‐Derived Prostate Cancer Organoids

For the establishment of prostate cancer organoids from patients who received ADT, the prostate cancer tissues were obtained from surgical specimens and confirmed with H&E staining by the Urology Center of the Shuguang Hospital. The tumor tissues were digested to establish the organoids. All organoids were cultured in the prostate cancer organoid‐specific MasterAim medium (100‐030, AIMINGMED, China). For the organoid viability assay, organoids were cultured in fresh prostate cancer organoid‐specific medium with specified dosages of L14‐8 or enzalutamide for 3 or 6 days. The organoids' morphology and viability were detected by brightfield imaging and staining with PI and Hoechst fluorescence dye, respectively. Ethical approval was obtained from the Ethics Committee of the Shuguang Hospital of Shanghai University of Traditional Chinese Medicine (Approval No. 2022121014702).

### Animal Studies

The male 4‐week‐old BALB/c nude mice (body weight was 20 ± 2 g) were obtained from the Shanghai Jihui Laboratory Animal Care Co. Ltd. (Shanghai, China) and housed in a temperature‐controlled room (24 ± 2 °C) with a 12‐h light/dark cycle. Following a 1‐week acclimatization period, the C4‐2B cells (1 × 10^6^/200 µL PBS) were injected subcutaneously into the mice. After another week, the animals were randomly assigned to three groups: Vehicle (saline), L14‐8 low dose (10 mg·kg^−1^·d^−1^), and L14‐8 high dose (20 mg·kg^−1^·d^−1^). The treatments were administered daily via the intraperitoneal injection, and the tumor sizes were measured every 2 days using calipers. After 25 days of treatment, the mice were euthanized for the evaluation of tumor mass, Ki67 staining, and potential organ toxicity. The histological examination involved fixing the hearts, livers, spleens, lungs, and kidneys in 4% paraformaldehyde, embedding in paraffin, sectioning, dewaxing in xylene, and staining with a Hematoxylin and Eosin Staining Kit (H&E) (60524ES60, Yeasen Biotechnology (Shanghai) Co. Ltd, China). The stained tissues were analyzed using a PreciPoint M8 Microscope and Scanner (Freising, Germany). All the procedures adhered to the animal ethics guidelines and were approved by the Animal Care and Use Committee at Shanghai University of Traditional Chinese Medicine (Ethics No. PZSHUTCM220627005).

### Network Pharmacology Analysis of L14‐8 Targets and Molecular Docking

The network pharmacology analysis of L14‐8 targets and molecular docking were conducted as previously described. Briefly, the SDF format of L14‐8 was uploaded to the SwissTargetPrediction portal (http://www.swissdock.ch/), and the top 50 targets with the highest probability were selected for further analysis (Table S5, Supporting Information). For molecular docking, the PDB format structures of PLK1 were downloaded from RCSB (https://www.rcsb.org/) and processed using PyMol. The docking simulations were performed using AutoDock Vina to evaluate the binding properties of the L14‐8 ligand to PLK1.

### Public Patient Cohorts Data Access and Analysis

The processed transcriptome data and corresponding patient information from TCGA‐PRAD,^[^ ^40^ ^]^ MSK,^[^ ^41^ ^]^ SUMM,^[^ ^42^ ^]^ neuroendocrine prostate cancer,^[^ ^43^ ^]^ and West Coast Dream Team^[^ ^44^ ^]^ were downloaded from cBioPortal (https://www.cbioportal.org/). The Kaplan‐Meier survival analysis, the tumor stage analysis, and the Pearson correlation analysis of gene expression were conducted using GraphPad Prism 10. The ChIP‐seq data were retrieved from Cistrome DB (http://cistrome.org) and visualized with the UCSC Browser (http://genome.ucsc.edu/).

### Cellular Thermal Shift Assay (CETSA)

CETSAs were conducted as previously described, with modifications.^[^ ^45^ ^]^ Briefly, the cells were treated with L14‐8 or DMSO for 6 h and then exposed to temperatures of 37, 41, 44, 47, 50, 53, 56, 59, 63, and 67 °C for 3 min each. The cells were then lysed using liquid nitrogen and a 25 °C heater for 3 cycles. The proteins were collected by centrifugation at 12000 rpm and 4 °C for 15 min, and western blot assays were performed to assess the expression of PLK1 and ACTB.

### Statistical Analysis

The data were analyzed using GraphPad Prism 10 software. The statistical significance was assessed using two‐sided unpaired *t*‐tests, and the results were presented as mean ± SD. All experiments were performed with at least three replicates independently. *p*‐values < 0.05 were considered statistically significant, with ^**^ indicating *p* < 0.01 and ^*^ indicating *p* < 0.05.

## Conflict of Interest

F.Y., W.Z., Y. Z., and X.S. are co‐inventors on a patent filed by Shanghai University of Traditional Chinese Medicine that relates to the research reported in this paper. The remaining authors declare no competing interests.

## Author Contributions

Y.Z., X.‐W.S, and N.Z. contributed equally to this work. Conceptualization was performed by F.W.Y. Methodology was developed by Y.Z., F.W.Y., and L.F.X. Investigation was carried out by X.W.S., Y.Z., N.Z., F.C.W., X.H.L., Y.A.W., and D.L.X. Visualization was done by X.W.S., Y.Z., and F.W.Y. Supervision was provided by F.W.Y. and L.F.X. Funding support was obtained by F.W.Y., L.F.X., and Y.Z. Writing of the original draft was done by F.W.Y. and Y.Z. Writing, reviewing, and editing were completed by X.W.S., N.Z., and X.H.L.

## Supporting information



Supporting Information

## Data Availability

The data that support the findings of this study are available from the corresponding author upon reasonable request.
