# Supplementary material for: Ezetimibe Engineered L14‐8 Suppresses Advanced Prostate Cancer by Activating PLK1/TP53‐SAT1‐Induced Ferroptosis
Source: Adv Sci (Weinh). 2025 Jun 19;12(29):e04192. doi: 10.1002/advs.202504192 (PMC12362806; doi:10.1002/advs.202504192)
Supplement: Supplementary file 1 — Supporting Information [file ADVS-12-e04192-s001.docx]

Supplementary Materials for

Ezetimibe engineered L14-8 suppresses advanced prostate cancer by activating PLK1/TP53-*SAT1*-induced ferroptosis

*Yu Zhang*†*, Xiao-wen Song*†*, Na Zhang*†*, Fan-chen Wu, Xue-hui Li, Yuang Wei, Dongliang Xu, Ling-fan Xu * , Fu-wen Yuan**

*Corresponding author: Fuwen Yuan (yuanfuwen@pku.edu.cn), Lingfan Xu ([ayfyxlf@163.com](mailto:ayfyxlf@163.com)).

†These authors contributed equally to this work.

**This Supplementary file includes:**

Supplementary Text

Figures S1 to S8

Tables S1 to S5

Supplementary Text

Supplementary Text

1. Construction of small-molecule library

The details for synthesizing diverse molecules was shown below:

- 1. Synthesis of N-tosylhydrazones

*N*-tosylhydrazones were prepared according to a reported procedure.^1^ To a stirred solution of *N*-tosylhydrazide (10 mmol) in MeOH (10 mL) at 60 ^o^C, ketone (1.0 equiv.) was added dropwise (or portion wise if solid). The reaction was completed within 0.5-3 h. After that, the solvent was removed directly under reduced pressure, and further purified by recrystallization or via silica gel chromatography (hexane:EtOAc, 2:1).

- 1. Synthesis of alkenes

A dry 5 mL Schlenk tube containing a stirring bar was charged with 0.2 mmol of N-tosylhydrazone (1.0 equiv.). After purging the flask three times under vacuum and three times under argon, it was charged with 0.3 mmol of DBU (1.5 equiv.) and anhydrous 2-MeTHF (1.0 mL), successively. The reaction was kept for 8 h under 40 W 456 nm Kessil lamp reaction setup (the progress can be monitored via TLC). Then, the resulting mixture is concentrated in vacuo. Products were purified via column chromatography with ethyl acetate and hexane as solvents. In addition, for products whose E/Z ratio can be obtained by ^1^H NMR.

- 1. Synthesis of pyrazoles and pyrazolines

Pyrazolines: A dry 5 mL Schlenk tube containing a stirring bar was charged with 0.2 mmol of N-tosylhydrazone (1.0 equiv.), 0.3 mmol of alkene (1.5 equiv.), 0.6 mmol of DBU (3.0 equiv.) and 0.6 mmol (3.0 equiv.) of H_2_O. After purging the flask for three times under vacuum and three times under argon, it was charged with 2-Methyltetrahydrofuran (1.0 mL). The reaction was kept for 16 h under 40 W Kessil lamp reaction setup (the progress can be monitored via TLC). Then, the resulting mixture underwent an aqueous workup (using distilled water; or brine in case of slurry phase separation) and was extracted three times with dichloromethane. The combined organic layers were dried over anhydrous Na_2_SO_4_, filtered and concentrated in vacuo. Products were purified via flash column chromatography with ethyl acetate and hexane as solvents.

Pyrazoles: A dry 5 mL Schlenk tube containing a stirring bar was charged with 0.2 mmol of *N*-tosylhydrazone (1.0 equiv.), 0.3 mmol of alkene (1.5 equiv.), 1.0 mmol of DBU (5.0 equiv.) and 0.6 mmol (3.0 equiv.) of H_2_O. After purging the flask for three times under vacuum and three times under argon, it was charged with 2-Methyltetrahydrofuran (1.0 mL). The reaction was kept for 16 h under 40 W Kessil lamp reaction setup (the progress can be monitored via TLC). Then, the resulting mixture underwent an aqueous workup (using distilled water; or brine in case of slurry phase separation) and was extracted three times with dichloromethane. The combined organic layers were dried over anhydrous Na_2_SO_4_, filtered and concentrated in vacuo. Products were purified via flash column chromatography with ethyl acetate and hexane as solvents.

- 1. Synthesis of cyclopropanes

A dry 5 mL Schlenk tube containing a stirring bar was charged with 0.1 mmol of *N*-tosylhydrazone (1.0 equiv.), 0.3 mmol of Cs_2_CO_3_ (3.0 equiv.) and 0.002 mmol (2 mol%) of 4CzIPN. After purging the flask for three times under vacuum and three times under argon, it was charged with 0.5 mmol of a (5.0 equiv.), toluene (1.0 mL), successively. The reaction was kept for 48 h under 40 W Kessil lamp reaction setup (the progress could be monitored via TLC). Then, the resulting mixture underwent an aqueous workup (using distilled water; or brine in case of slurry phase separation) and was extracted three times with ethyl acetate. The combined organic layers were dried over anhydrous Na_2_SO_4_, filtered and concentrated in vacuo. Products were purified via chromatography with ethyl acetate and hexane as solvents.

- 1. Synthesis of ethers and thioethers

Thioethers: A dry 5 mL Schlenk tube containing a stirring bar was charged with 0.1 mmol of N-tosylhydrazone (1.0 equiv.), 0.1 mmol of K_3_PO_4_ (1.0 equiv.). After purging the flask for three times under vacuum and three times under argon, it was charged with 0.5 mmol of b (5.0 equiv.), PhCF_3_ (1.0 mL), successively. The reaction was kept for 1 h under 40 W Kessil lamp reaction setup (the progress could be monitored via TLC). Then, the resulting mixture underwent an aqueous workup (using distilled water; or brine in case of slurry phase separation) and was extracted three times with ethyl acetate. The combined organic layers were dried over anhydrous Na_2_SO_4_, filtered and concentrated in vacuo. Products were purified via chromatography with ethyl acetate and hexane as solvents.

Ethers: A dry 5 mL Schlenk tube containing a stirring bar was charged with N-tosylhydrazone (0.2 mmol, 1.0 equiv) and K_2_CO_3_ (0.4 mmol, 2.0 equiv). After purging the flask three times under vacuum and three times under argon, it was charged with 2.0 mmol of alcohols (10.0 equiv) and anhydrous 1,4-Dioxane (0.8 mL), successively. The reaction was kept for 16 h under 40 W 427nm Kessil lamp reaction setup (the progress can be monitored via TLC). Then, the resulting mixture is concentrated in the vacuo. Products were purified via column chromatography with ethyl acetate and hexane as solvents. In addition, for products whose D/R ratio can be obtained by 1H NMR.

1.6 Synthesis of amines

A dry 5 mL Schlenk tube containing a stirring bar was charged with 0.2 mmol of *N*-tosylhydrazone (1.0 equiv.), 1.0 mmol of arylamine (5.0 equiv.). After purging the flask for three times under vacuum and three times under argon, it was charged with 0.3 mmol of DBN (1.5 equiv.), DCM (2.0 mL), successively. The reaction was kept for 6 h under 40 W Kessil lamp reaction setup (the progress can be monitored via TLC). Then, the resulting mixture underwent an aqueous workup (using distilled water; or brine in case of slurry phase separation) and was extracted three times with ethyl acetate. The combined organic layers were dried over anhydrous Na_2_SO_4_, filtered and concentrated in vacuo. Products were purified via Flash chromatography chromatography with ethyl acetate and hexane as solvents.

- 1. Structural optimization of L14.

A dry 50 mL round-bottom flask containing a stirring bar was charged with alcohols compounds (6.5 mmol, 1.0 equiv.), triethylamine (9.8 mmol, 1.5 equiv.) and 25 mL of dichloromethane at room temperature. Then, acyl chloride (7.8 mmol, 1.2 equiv.) was added at 0 °C. The reaction was carried out under room temperature for 24 h (the process can be monitored by TLC). The resulting mixture was then subjected to aqueous treatment (using distilled water; or brine in case of slurry phase separation) and extracted three times with dichloromethane. The combined organic layer was dried over anhydrous sodium sulfate, filtered, and concentrated in a vacuum. The product was purified by flash chromatography to give a colorless oil (EA: hexane=1:3).

Note: It is necessary to add acyl chloride at 0 °C with the ice bath, and the result of the high temperature is that the system produces a lot of impurities, which leads to reduced conversion and yield. It is necessary to change the ratio of the acyl chloride if double substitution product was synthesized. In contrast, there will be a lot of impurities. Another feasible approach is to use single substituents to re-react and achieve a large number of double substitution products.

(S)-1-(4-fluorophenyl)-3-((3R,4S)-1-(4-fluorophenyl)-2-oxo-4-(4-(pent-4-enoyloxy)phenyl) azetidin-3-yl) propyl pent-4-enoate (**L14**)

^1^H NMR (600 MHz, CDCl_3_) δ 7.31 (d, J = 8.6 Hz, 2H), 7.28 – 7.25 (m, 2H), 7.21 (dd, J = 9.1, 4.6 Hz, 2H), 7.10 (d, J = 8.5 Hz, 2H), 7.01 (t, J = 8.6 Hz, 2H), 6.96 – 6.89 (m, 2H), 5.89 (dd, J = 17.0, 10.4 Hz, 1H), 5.77 (dd, J = 17.0, 10.4 Hz, 1H), 5.71 (t, J = 6.8 Hz, 1H), 5.18 – 4.88 (m, 4H), 4.59 (d, J = 2.4 Hz, 1H), 3.10 – 3.00 (m, 1H), 2.67 (t, J = 7.4 Hz, 2H), 2.54 – 2.47 (m, 2H), 2.45 – 2.38 (m, 2H), 2.35 (t, J = 6.4 Hz, 2H), 2.09 – 1.95 (m, 2H), 1.94 – 1.74 (m, 2H).

^13^C NMR (151 MHz, CDCl_3_) δ 172.1, 171.4, 166.7, 162.4 (d, J = 246.8 Hz), 159.0 (d, J = 243.6 Hz)，, 150.8, 136.4, 136.1, 135.7 (d, J = 3.2 Hz), 134.9, 133.7 (d, J = 2.7 Hz), 128.2 (d, J = 8.2 Hz), 126.8, 122.5, 118.3 (d, J = 7.9 Hz), 116.0, 116.0, 115.8, 115.6 (d, J = 5.1 Hz), 115.4, 74.8, 60.7, 60.2, 33.7 (d, J = 9.4 Hz), 28.8 (d, J = 7.1 Hz), 24.9.

ESI-MS: calcd. for C_34_H_33_F_2_NO_5_ [M+H]^+^: 574.2400, found: 574.2408.

4-((2S,3R)-1-(4-fluorophenyl)-3-((S)-3-(4-fluorophenyl)-3-hydroxypropyl)-4-oxoazetidin-2-yl) phenyl pent-4-enoate (**L14-1**)

^1^H NMR (600 MHz, CDCl_3_) δ 7.35 – 7.25 (m, 4H), 7.21 (dd, J = 9.0, 4.6 Hz, 2H), 7.09 (d, J = 8.4 Hz, 2H), 7.01 (t, J = 8.6 Hz, 2H), 6.93 (t, J = 8.6 Hz, 2H), 5.88 (dd, J = 17.0, 10.4 Hz, 1H), 5.19 – 5.01 (m, 2H), 4.77 – 4.68 (m, 1H), 4.62 (d, J = 2.3 Hz, 1H), 3.08 (t, J = 7.4 Hz, 1H), 2.66 (t, J = 7.4 Hz, 2H), 2.50 (t, J = 7.0 Hz, 2H), 2.05 – 1.81 (m, 4H).

^13^C NMR (151 MHz, CDCl_3_) δ 171.4, 167.4, 162.1 (d, J = 245.5 Hz), 159.0 (d, J = 243.7 Hz), 150.7, 140.0 (d, J = 3.0 Hz), 136.1, 134.9, 133.6 (d, J = 2.7 Hz), 127.3 (d, J = 8.0 Hz), 126.8, 122.4, 118.3 (d, J = 7.9 Hz), 116.0, 115.9 (d, J = 22.6 Hz), 115.3 (d, J = 21.3 Hz), 73.0, 60.8, 60.3, 36.5, 33.5, 28.8, 24.9.

ESI-MS: calcd. for C_29_H_27_F_2_NO_4_ [M+H]^+^: 492.1981, found: 492.1987.

4-((2S,3R)-1-(4-fluorophenyl)-3-((S)-3-(4-fluorophenyl)-3-hydroxypropyl)-4-oxoazetidin-2-yl) phenyl (E)-but-2-enoate (**L14-2**)

^1^H NMR (400 MHz, CDCl_3_) δ 7.37 – 7.27 (m, 4H), 7.23 (dd, J = 9.1, 4.7 Hz, 2H), 7.13 (d, J = 8.6 Hz, 2H), 7.02 (t, J = 8.7 Hz, 2H), 6.94 (dd, J = 9.3, 8.1 Hz, 2H), 6.04 (dd, J = 15.5, 1.8 Hz, 1H), 4.72 (s, 1H), 4.63 (d, J = 2.4 Hz, 1H), 3.09 (dt, J = 7.7, 3.6 Hz, 1H), 2.31 – 2.17 (m, 1H), 1.97 (dd, J = 6.9, 1.7 Hz, 6H), 1.60 (s, 1H).

ESI-MS: calcd. for C_28_H_25_F_2_NO_4_ [M+H]^+^: 478.1824, found: 478.1832.

4-((2S,3R)-1-(4-fluorophenyl)-3-((S)-3-(4-fluorophenyl)-3-hydroxypropyl)-4-oxoazetidin-2-yl) phenyl 5-chlorothiophene-2-carboxylate (**L14-3**)

^1^H NMR (600 MHz, CDCl_3_) δ 7.76 (d, J = 4.0 Hz, 1H), 7.37 (d, J = 8.6 Hz, 2H), 7.29 (dd, J = 8.6, 5.5 Hz, 2H), 7.25 – 7.20 (m, 4H), 7.05 – 6.99 (m, 3H), 6.97 – 6.90 (m, 2H), 4.77 – 4.69 (m, 1H), 4.65 (d, J = 2.3 Hz, 1H), 3.10 (td, J = 7.5, 2.4 Hz, 1H), 2.33 (s, 1H), 2.03 – 1.77 (m, 4H).

^13^C NMR (151 MHz, CDCl_3_) δ 167.3, 162.2 (d, J = 245.8 Hz)., 159.4, 159.0 (d, J = 243.7 Hz)., 150.4, 140.0 (d, J = 3.2 Hz), 139.0, 135.4, 134.5, 133.7 (d, J = 2.7 Hz), 130.5, 127.6, 127.4 (d, J = 8.0 Hz), 127.0, 122.5, 118.3 (d, J = 7.9 Hz), 115.9 (d, J = 22.6 Hz), 115.4 (d, J = 21.4 Hz), 73.1, 60.8, 60.4, 36.6, 25.0, 21.0, 14.2.

ESI-MS: calcd. for C_29_H_22_ClF_2_NO_4_S [M+H]^+^: 554.0999, found: 555.0005.

4-((2S,3R)-1-(4-fluorophenyl)-3-((S)-3-(4-fluorophenyl)-3-hydroxypropyl)-4-oxoazetidin-2-yl) phenyl cyclobutanecarboxylate (**L14-4**)

^1^H NMR (600 MHz, CDCl_3_) δ 7.36 – 7.27 (m, 4H), 7.22 (dd, J = 9.0, 4.6 Hz, 2H), 7.10 (d, J = 8.5 Hz, 2H), 7.02 (t, J = 8.6 Hz, 2H), 6.93 (t, J = 8.7 Hz, 2H), 4.80 – 4.67 (m, 1H), 4.62 (d, J = 2.4 Hz, 1H), 3.38 (td, J = 8.5, 1.1 Hz, 1H), 3.14 – 3.00 (m, 1H), 2.50 – 2.37 (m, 2H), 2.37 – 2.16 (m, 3H), 2.13 – 1.80 (m, 6H).

^13^C NMR (151 MHz, CDCl_3_) δ 173.8, 167.4, δ 162.2 (d, J = 245.9 Hz)., 159.0 (d, J = 243.7 Hz)., 151.0, 140.0 (d, J = 3.3 Hz), 134.8, 133.70 (d, J = 2.7 Hz), 127.4 (d, J = 8.1 Hz), 126.8, 122.4, 118.4 (d, J = 7.7 Hz). 115.9 (d, J = 22.7 Hz), 115.4 (d, J = 21.2 Hz), 73.1, 60.9, 60.4, 38.1, 36.6, 25.3, 25.0, 18.4.

ESI-MS: calcd. for C_29_H_27_F_2_NO_4_ [M+H]^+^: 492.1981, found: 492.1989.

4-((2S,3R)-1-(4-fluorophenyl)-3-((S)-3-(4-fluorophenyl)-3-hydroxypropyl)-4-oxoazetidin-2-yl) phenyl cyclopropanecarboxylate (**L14-5**)

^1^H NMR (600 MHz, CDCl_3_) δ 7.33 (d, J = 8.6 Hz, 2H), 7.31 – 7.26 (m, 2H), 7.23 (dd, J = 9.0, 4.6 Hz, 2H), 7.12 (s, 2H), 7.02 (t, J = 8.7 Hz, 2H), 6.94 (t, J = 8.7 Hz, 2H), 4.76 – 4.67 (m, 1H), 4.64 (d, J = 2.4 Hz, 1H), 3.11 – 3.06 (m, 1H), 2.07 – 1.78 (m, 5H), 1.20 – 1.16 (m, 2H), 1.05 (dd, J = 8.0, 3.2 Hz, 2H).

^13^C NMR (151 MHz, CDCl_3_) δ 173.3, 167.4, 162.1 (d, J = 245.5 Hz), 159.0 (d, J = 243.6 Hz), 150.8, 140.0 (d, J = 3.2 Hz), 134.7, 133.6 (d, J = 2.7 Hz), 127.3 (d, J = 8.1 Hz), 126.8, 122.4, 118.3 (d, J = 7.8 Hz), 115.8 (d, J = 22.8 Hz), 115.2 (d, J = 21.3 Hz), 72.9, 60.8, 60.2, 36.5, 24.9, 12.9, 9.3, 8.9.

ESI-MS: calcd. for C_28_H_25_F_2_NO_4_ [M+H]^+^: 478.1824, found: 478.1827.

4-((2S,3R)-1-(4-fluorophenyl)-3-((S)-3-(4-fluorophenyl)-3-hydroxypropyl)-4-oxoazetidin-2-yl) phenyl 4-methylpentanoate (**L14-6**)

^1^H NMR (600 MHz, CDCl_3_) δ 7.32 (d, J = 8.5 Hz, 2H), 7.29 (dd, J = 8.5, 5.5 Hz, 2H), 7.22 (dd, J = 9.0, 4.6 Hz, 2H), 7.09 (d, J = 8.5 Hz, 2H), 7.02 (t, J = 8.7 Hz, 2H), 6.93 (t, J = 8.7 Hz, 2H), 4.75 – 4.68 (m, 1H), 4.62 (d, J = 2.4 Hz, 1H), 3.09 (td, J = 7.4, 2.4 Hz, 1H), 2.56 (dd, J = 8.1, 6.9 Hz, 2H), 2.04 – 1.84 (m, 4H), 1.71 – 1.57 (m, 4H), 0.95 (d, J = 6.1 Hz, 6H).

^13^C NMR (151 MHz, CDCl_3_) δ 172.4, 167.3, 162.2 (d, J = 245.5 Hz), 159.0 (d, J = 243.5 Hz), 150.9, 140.0 (d, J = 3.2 Hz), 134.9, 133.7 (d, J = 2.7 Hz), 127.4 (d, J = 8.2 Hz), 126.9, 122.5, 118.4 (d, J = 7.8 Hz), 115.9 (d, J = 22.6 Hz), 115.4 (d, J = 21.2 Hz), 73.1, 60.9, 60.4, 36.5, 33.6, 32.4, 27.7, 25.0, 22.2.

ESI-MS: calcd. for C_30_H_31_F_2_NO_4_ [M+H]^+^: 508.2294, found: 508.2283.

4-((2S,3R)-1-(4-fluorophenyl)-3-((S)-3-(4-fluorophenyl)-3-hydroxypropyl)-4-oxoazetidin-2-yl) phenyl pentanoate (**L14-7**)

^1^H NMR (600 MHz, CDCl_3_) δ 7.34 (d, J = 8.6 Hz, 2H), 7.30 (dd, J = 8.5, 5.5 Hz, 2H), 7.24 (dd, J = 9.0, 4.6 Hz, 2H), 7.11 (d, J = 8.5 Hz, 2H), 7.03 (t, J = 8.7 Hz, 2H), 6.95 (t, J = 8.6 Hz, 2H), 4.79 – 4.68 (m, 1H), 4.64 (d, J = 2.3 Hz, 1H), 3.10 (td, J = 7.4, 2.4 Hz, 1H), 2.58 (t, J = 7.5 Hz, 2H), 2.04 – 1.86 (m, 4H), 1.75 (p, J = 7.5 Hz, 2H), 1.46 (h, J = 7.4 Hz, 2H), 0.99 (t, J = 7.4 Hz, 3H).

^13^C NMR (151 MHz, CDCl_3_) δ 172.2, 167.4, 162.1 (d, J = 245.7 Hz), 159.0 (d, J = 243.7 Hz), 150.8, 140.0 (d, J = 3.2 Hz), 134.8, 133.6 (d, J = 2.7 Hz), 127.3 (d, J = 8.0 Hz), 126.8, 122.4, 118.3 (d, J = 7.8 Hz), 115.8 (d, J = 22.6 Hz), 115.3 (d, J = 21.4 Hz), 73.0, 60.8, 60.3, 36.5, 34.0, 26.9, 24.9, 22.2, 13.7.

ESI-MS: calcd. for C_29_H_29_F_2_NO_4_ [M+H]^+^: 494.2137, found: 494.2143.

4-((2S,3R)-3-((S)-3-(acryloyloxy)-3-(4-fluorophenyl)propyl)-1-(4-fluorophenyl)-4-oxoazetidin-2-yl) phenyl acrylate (**L14-8**)

^1^H NMR (500 MHz, CDCl_3_) δ 7.34 (d, J = 8.6 Hz, 2H), 7.29 (dd, J = 8.8, 5.2 Hz, 2H), 7.22 (dd, J = 9.1, 4.7 Hz, 2H), 7.16 (d, J = 8.6 Hz, 2H), 7.02 (t, J = 8.7 Hz, 2H), 6.93 (dd, J = 9.1, 8.3 Hz, 2H), 6.61 (dd, J = 17.3, 1.2 Hz, 1H), 6.41 (dd, J = 17.3, 1.4 Hz, 1H), 6.32 (dd, J = 17.3, 10.5 Hz, 1H), 6.12 (dd, J = 17.3, 10.4 Hz, 1H), 6.04 (dd, J = 10.5, 1.2 Hz, 1H), 5.85 (dd, J = 10.4, 1.4 Hz, 1H), 5.79 (t, J = 6.8 Hz, 1H), 4.61 (d, J = 2.4 Hz, 1H), 3.08 (td, J = 8.4, 7.9, 2.4 Hz, 1H), 2.12 – 2.03 (m, 2H), 1.94 – 1.84 (m, 2H).

^13^C NMR (126 MHz, CDCl_3_) δ 166.7, 165.3, 164.3, 163.4, 162.4 (d, J = 246.8 Hz), 159.0 (d, J = 243.6 Hz), 150.7, 135.5, 134.9, 133.6, 133.0, 131.3, 128.2, 128.2, 128.2, 127.6, 126.9, 122.5, 118.3 (d, J = 7.7 Hz), 115.9 (d, J = 22.9 Hz), 115.5 (d, J = 21.5 Hz), 75.0, 60.7, 60.1, 33.6, 24.9.

ESI-MS: calcd. for C_30_H_25_F_2_NO_5_ [M+H]^+^: 518.1774, found: 518.1785.

4-((2S,3R)-3-((S)-3-((cyclobutanecarbonyl)oxy)-3-(4-fluorophenyl)propyl)-1-(4-fluorophenyl)-4-oxoazetidin-2-yl)phenyl cyclobutanecarboxylate (**L14-9**)

^1^H NMR (600 MHz, CDCl_3_) δ 7.31 (d, J = 8.5 Hz, 2H), 7.28 – 7.24 (m, 2H), 7.21 (dd, J = 9.1, 4.6 Hz, 2H), 7.10 (d, J = 8.5 Hz, 2H), 7.01 (t, J = 8.7 Hz, 2H), 6.92 (t, J = 8.7 Hz, 2H), 5.69 (t, J = 6.8 Hz, 1H), 4.59 (d, J = 2.3 Hz, 1H), 3.38 (t, J = 8.5 Hz, 1H), 3.18 – 3.09 (m, 1H), 3.07 (td, J = 7.8, 2.4 Hz, 1H), 2.41 (d, J = 8.5 Hz, 2H), 2.37 – 2.27 (m, 2H), 2.19 (d, J = 8.5 Hz, 4H), 2.10 – 1.92 (m, 5H), 1.87 (d, J = 7.1 Hz, 3H).

^13^C NMR (151 MHz, CDCl_3_) δ 174.5, 173.7, 166.7, 162.4 (d, J = 246.5 Hz), 159.0 (d, J = 243.6 Hz), 151.0, 135.9 (d, J = 3.2 Hz), 134.7, 133.7 (d, J = 2.6 Hz), 128.1 (d, J = 8.2 Hz), 126.8, 122.5, 118.3 (d, J = 7.7 Hz), 115.9 (d, J = 22.6 Hz), 115.5 (d, J = 21.5 Hz), 74.5, 60.7, 60.2, 38.1 (d, J = 4.4 Hz), 33.7, 25.3 (d, J = 2.1 Hz), 25.1, 25.0, 24.9, 18.4 (d, J = 5.9 Hz).

ESI-MS: calcd. for C_34_H_33_F_2_NO_5_ [M+H]^+^: 574.2400, found: 574.2405.

4-((2S,3R)-3-((S)-3-((cyclopropanecarbonyl)oxy)-3-(4-fluorophenyl) propyl)-1-(4-fluorophenyl)-4-oxoazetidin-2-yl) phenyl cyclopropanecarboxylate (**L14-10**)

^1^H NMR (600 MHz, CDCl_3_) δ 7.31 (d, J = 8.3 Hz, 2H), 7.29 – 7.24 (m, 2H), 7.21 (dd, J = 8.9, 4.6 Hz, 2H), 7.11 (d, J = 8.3 Hz, 2H), 7.02 (t, J = 8.5 Hz, 2H), 6.92 (t, J = 8.6 Hz, 2H), 5.70 (t, J = 6.8 Hz, 1H), 4.59 (d, J = 2.3 Hz, 1H), 3.06 (td, J = 7.8, 2.4 Hz, 1H), 2.03 (dt, J = 16.5, 6.8 Hz, 2H), 1.85 (ddd, J = 15.8, 9.0, 4.5 Hz, 2H), 1.62 (dd, J = 8.3, 3.9 Hz, 2H), 1.19 – 1.13 (m, 2H), 1.03 (dd, J = 7.9, 3.3 Hz, 2H), 1.01 – 0.91 (m, 2H), 0.86 (ddt, J = 11.4, 8.4, 5.8 Hz, 2H).

^13^C NMR (151 MHz, CDCl_3_) δ 174.0, 173.3, 166.7, 162.4 (d, J = 246.5 Hz), 159.0 (d, J = 243.4 Hz), 150.9, 135.8 (d, J = 3.2 Hz), 134.7, 133.7, 128.1 (d, J = 8.2 Hz), 126.8, 122.5, 118.3 (d, J = 7.9 Hz), 115.9, 115.8, 115.6, 115.4, 74.7, 60.4 (d, J = 79.2 Hz), 33.7, 24.9, 13.0 (d, J = 2.7 Hz), 9.4, 8.6 (d, J = 6.5 Hz).

ESI-MS: calcd. for C_32_H_29_F_2_NO_5_ [M+H]^+^: 546.2087, found: 546.2096.

(S)-1-(4-fluorophenyl)-3-((3R,4S)-1-(4-fluorophenyl)-2-oxo-4-(4-(pentanoyloxy) phenyl) azetidin-3-yl) propyl pentanoate (**L14-11**)

^1^H NMR (600 MHz, CDCl_3_) δ 7.32 (d, J = 8.3 Hz, 2H), 7.26 (dd, *J* = 8.4, 5.4 Hz, 2H), 7.21 (dd, J = 8.9, 4.6 Hz, 2H), 7.10 (d, J = 8.4 Hz, 2H), 7.01 (t, J = 8.6 Hz, 2H), 6.92 (t, J = 8.6 Hz, 2H), 5.70 (t, J = 6.8 Hz, 1H), 4.59 (d, J = 2.4 Hz, 1H), 3.06 (t, J = 6.6 Hz, 1H), 2.56 (t, J = 7.5 Hz, 2H), 2.31 (td, J = 7.5, 4.0 Hz, 2H), 2.03 (dd, J = 11.3, 6.0 Hz, 2H), 1.85 (td, J = 16.0, 14.4, 6.3 Hz, 2H), 1.73 (p, J = 7.5 Hz, 2H), 1.58 (p, J = 7.6 Hz, 2H), 1.44 (q, J = 7.5 Hz, 2H), 1.30 (q, J = 7.4 Hz, 2H), 0.96 (t, J = 7.4 Hz, 3H), 0.89 (t, J = 7.4 Hz, 3H).

^13^C NMR (151 MHz, CDCl_3_) δ 172.9, 172.1, 166.7, 162.4 (d, J = 246.5 Hz), 159.0 (d, J = 243.6 Hz), 150.9, 135.8, 134.8, 133.6 (d, J = 2.7 Hz), 128.1 (d, J = 8.1 Hz), 126.8, 122.5, 118.3 (d, J = 7.7 Hz), 115.8 (d, J = 22.6 Hz), 115.5 (d, J = 21.5 Hz), 74.5, 60.4 (d, J = 78.0 Hz), 34.1 (d, J = 15.9 Hz), 33.6, 26.9, 24.9, 22.2, 13.6 (d, J = 4.5 Hz).

ESI-MS: calcd. for C_34_H_37_F_2_NO_5_ [M+H]^+^: 578.2713, found: 578.2709.

4-((2S,3R)-1-(4-fluorophenyl)-3-((S)-3-(4-fluorophenyl)-3-(isopentyloxy) propyl)-4-oxoazetidin-2-yl) phenyl 4-methylpentanoate (**L14-12**)

^1^H NMR (600 MHz, CDCl_3_) δ 7.31 (d, J = 8.5 Hz, 2H), 7.27 (s, 2H), 7.21 (dd, J = 9.0, 4.6 Hz, 2H), 7.10 (d, *J* = 8.4 Hz, 2H), 7.01 (t, *J* = 8.6 Hz, 2H), 6.93 (t, J = 8.6 Hz, 2H), 5.70 (t, J = 6.8 Hz, 1H), 4.59 (d, J = 2.4 Hz, 1H), 3.06 (td, J = 7.8, 2.4 Hz, 1H), 2.59 – 2.52 (m, 2H), 2.37 – 2.26 (m, 2H), 2.02 (d, J = 6.6 Hz, 2H), 1.86 (d, J = 10.4 Hz, 2H), 1.65 (t, J = 6.7 Hz, 2H), 1.57 (s, 2H), 1.49 (t, J = 7.6 Hz, 2H), 0.95 (d, J = 6.0 Hz, 6H), 0.87 (dd, J = 6.2, 2.9 Hz, 6H).

^13^C NMR (151 MHz, CDCl_3_) δ 173.2, 172.4, 166.7, 162.4 (d, J = 246.6 Hz), 159.0 (d, J = 243.6 Hz), 150.9, 135.8 (d, J = 3.3 Hz), 134.8, 133.7 (d, J = 2.6 Hz), 128.2 (d, J = 8.2 Hz), 126.8, 122.5, 118.3 (d, J = 8.0 Hz), 116.0, 115.8, 115.6, 115.4, 105.0, 74.6, 60.4 (d, J = 79.7 Hz), 33.6 (d, J = 4.1 Hz), 32.5, 32.4, 27.7, 27.6, 24.9, 22.23 – 22.15 (m).

ESI-MS: calcd. for C_35_H_41_F_2_NO_4_ [M+H]^+^: 578.3076, found: 578.3082.

**(S)-1-(4-fluorophenyl)-3-((3R,4S)-1-(4-fluorophenyl)-2-oxo-4-(4-(pent-4-enoyloxy)phenyl) azetidin-3-yl)propyl pent-4-enoate (L14)**

^1^H NMR spectrum in CDCl_3_.

^13^C NMR spectrum in CDCl_3_.

**4-((2S,3R)-1-(4-fluorophenyl)-3-((S)-3-(4-fluorophenyl)-3-hydroxypropyl)-4-oxoazetidin-2-yl)phenyl pent-4-enoate (L14-1)**

^1^H NMR spectrum in CDCl_3_.

^13^C NMR spectrum in CDCl_3_.

**4-((2S,3R)-1-(4-fluorophenyl)-3-((S)-3-(4-fluorophenyl)-3-hydroxypropyl)-4-oxoazetidin-2-yl)phenyl (E)-but-2-enoate (L14-2)**

^1^H NMR spectrum in CDCl_3_.

**4-((2S,3R)-1-(4-fluorophenyl)-3-((S)-3-(4-fluorophenyl)-3-hydroxypropyl)-4-oxoazetidin-2-yl)phenyl 5-chlorothiophene-2-carboxylate (L14-3)**

^1^H NMR spectrum in CDCl_3_.

^13^C NMR spectrum in CDCl_3_.

**4-((2S,3R)-1-(4-fluorophenyl)-3-((S)-3-(4-fluorophenyl)-3-hydroxypropyl)-4-oxoazetidin-2-yl)phenyl cyclobutanecarboxylate (L14-4)**

^1^H NMR spectrum in CDCl_3_.

^13^C NMR spectrum in CDCl_3_.

**4-((2S,3R)-1-(4-fluorophenyl)-3-((S)-3-(4-fluorophenyl)-3-hydroxypropyl)-4-oxoazetidin-2-yl)phenyl cyclopropanecarboxylate (L14-5)**

^1^H NMR spectrum in CDCl_3_.

^13^C NMR spectrum in CDCl_3_.

**4-((2S,3R)-1-(4-fluorophenyl)-3-((S)-3-(4-fluorophenyl)-3-hydroxypropyl)-4-oxoazetidin-2-yl) phenyl 4-methylpentanoate (L14-6)**

^1^H NMR spectrum in CDCl_3_.

^13^C NMR spectrum in CDCl_3_.

**4-((2S,3R)-1-(4-fluorophenyl)-3-((S)-3-(4-fluorophenyl)-3-hydroxypropyl)-4-oxoazetidin-2-yl) phenyl pentanoate (L14-7)**

^1^H NMR spectrum in CDCl_3_.

^13^C NMR spectrum in CDCl_3_.

**4-((2S,3R)-3-((S)-3-(acryloyloxy)-3-(4-fluorophenyl)propyl)-1-(4-fluorophenyl)-4-oxoazetidin-2-yl)phenyl acrylate (L14-8)**

^1^H NMR spectrum in CDCl_3_.

^13^C NMR spectrum in CDCl_3_.

**4-((2S,3R)-3-((S)-3-((cyclobutanecarbonyl)oxy)-3-(4-fluorophenyl)propyl)-1-(4-fluorophenyl)-4-oxoazetidin-2-yl)phenyl cyclobutanecarboxylate (L14-9)**

^1^H NMR spectrum in CDCl_3_.

^13^C NMR spectrum in CDCl_3_.

**4-((2S,3R)-3-((S)-3-((cyclopropanecarbonyl)oxy)-3-(4-fluorophenyl)propyl)-1-(4-fluorophenyl)-4-oxoazetidin-2-yl)phenyl cyclopropanecarboxylate (L14-10)**

^1^H NMR spectrum in CDCl_3_.

^13^C NMR spectrum in CDCl_3_.

**(S)-1-(4-fluorophenyl)-3-((3R,4S)-1-(4-fluorophenyl)-2-oxo-4-(4-(pentanoyloxy)phenyl)azetidin-3-yl)propyl pentanoate（L14-11）**

^1^H NMR spectrum in CDCl_3_.

^13^C NMR spectrum in CDCl_3_.

**4-((2S,3R)-1-(4-fluorophenyl)-3-((S)-3-(4-fluorophenyl)-3-(isopentyloxy)propyl)-4-oxoazetidin-2-yl)phenyl 4-methylpentanoate (L14-12)**

^1^H NMR spectrum in CDCl_3_.

^13^C NMR spectrum in CDCl_3_.

**
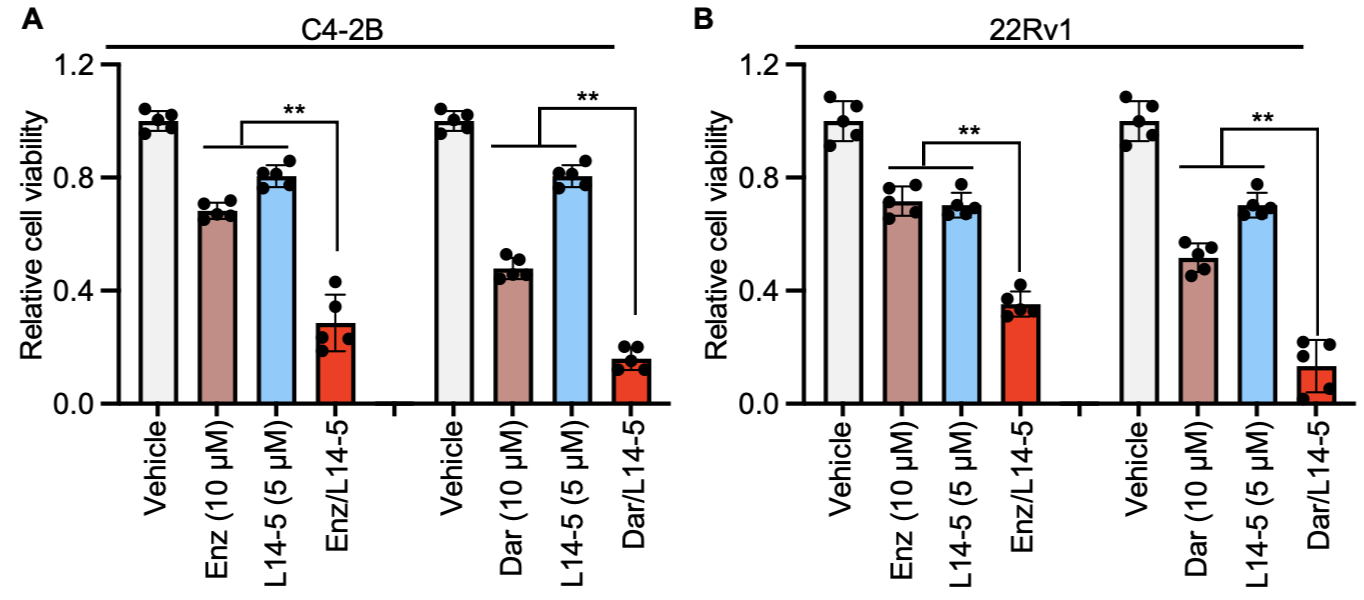
**

**Figure S1. L14-8 sensitizes CRPC cells to AR antagonists.** (A) Cell growth of CRPC cells C4-2B (A) and 22Rv1 (B) after treated with L14-8 and enzalutamide (Enz) or darolutamide (Dar) alone at the indicated dosages or combination were measured after 3 days of treatments (n=5). **, p <0.01.

**Figure S2. L14-8 treatment induced death of prostate cancer organoids derived from prostate cancer patients who received ADT compared to AR antagonists.** (A) Schematic illustrates the procedures for the establishment of prostate cancer patient-derived organoids. (B and C) Patient-derived prostate cancer organoids were treated with indicated agents, and the morphology and viability were detected by brightfield imaging and staining with PI (red, dead) and Hoechst (blue, alive) fluorescence dye, respectively. The relative organoid viability was statistically analyzed as shown in the right panel (I), n=3. ns, not significant, **, p < 0.01.

**Figure S3. L14-8 treatment induced decreased lipid peroxidation in prostate cancer cells.** Flow cytometry analysis and (A) fluorescence microscopy images (B) of living cells stained with C11- BODIPY581/591 after cells were treated with vehicle, L14-8, and ferroptosis repressor Ferr-1.

**Figure S4. L14-8 induced apoptosis in a dosage-dependent manner in different prostate cancer cells.** (A-D) Cellular apoptosis of C4-2B, 22Rv1, and PC3 (B) after being treated with different doses of L14-8 were stained with PI and Annexin-V and were analyzed with flow cytometry. (E) CCK-8 assays were conducted to evaluate the growth inhibition of L14-8 on PC3 cells. (F and G) Cellular apoptosis of PC3 after being treated with different doses of L14-8 were stained with PI and Annexin-V and were analyzed with flow cytometry (n=3). ns, not significant, **, P<0.01.

**
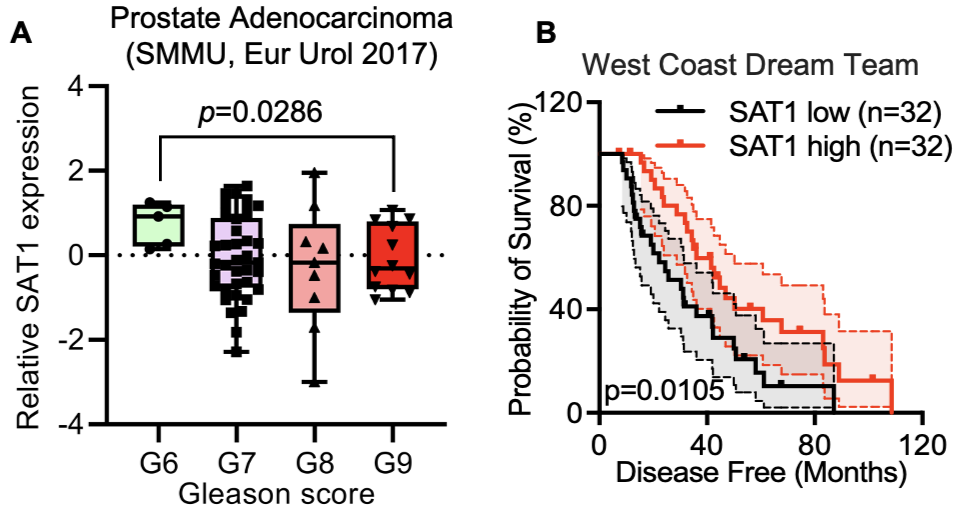
**

**Figure S5. SAT1 expression is correlated with Gleason score and patient survival in different prostate cancer cohorts.** (A and B) The expression correlation of SAT1 with Glasson score (A) and patient survival (B) in SUMMU and MSK prostate cancer cohort.

**
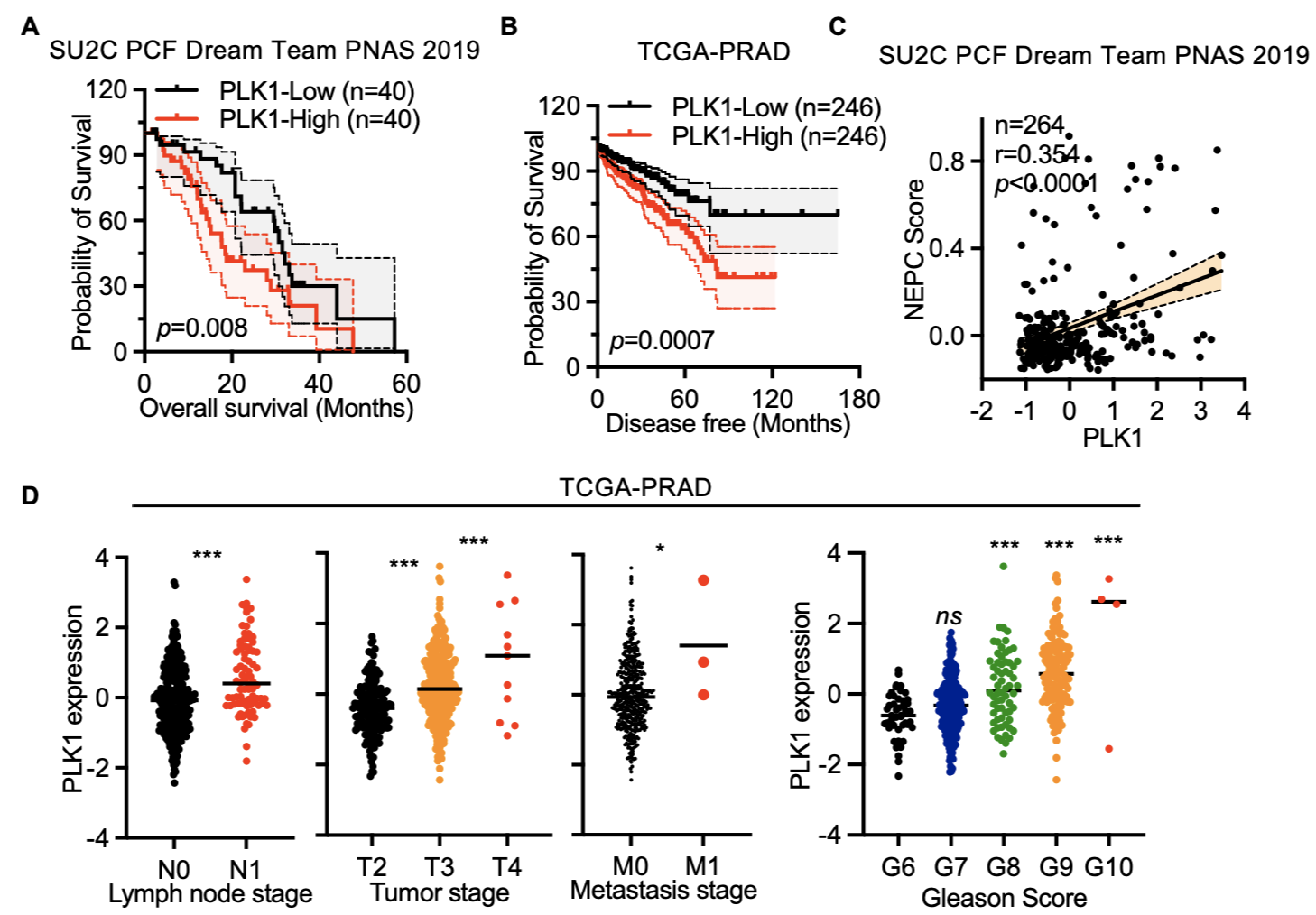
**

**Figure S6. PLK1 showed a prognostic indicator in different prostate cancer cohorts.** (A and B) Survival analysis of PKL1 and patient survival in SU2C (A) and TCGA-PRAD (B) cohorts. (C) PKL1 expression is positively correlated with NEPC score in the SU2C CRPC patient cohort. (D) The expression of PLK1 is higher in the late stages of prostate cancer in TCGA-PRAD cohort.

**Figure S7. The impact of L14-8 on PLK1 and TP53 transcription.** (A and B) C4-2B (A) and 22Rv1 (B) cells were treated with vehicle or indicated dosages of L14-8. After 48 h of treatment, the total RNA was then collected and reversed for mRNA expression. ns, not significant.

**Figure S8. The predicted targets and regulated genes of L14-8 and ezetimibe.** (A) The predicted targets of L14-8 and ezetimibe retrieved from SwissTargetPrediction platform. (B) The overlap of L14-8 and ezetimibe differentially regulated genes determined by transcriptome analysis. The RNA-seq of L14-8 differentially regulated genes were conducted in this study and the transcriptome analysis of ezetimibe differentially regulated genes were retrieved from previous study (*1*). (C) heatmap of transcriptome changes after L14-8 treatment. Labeled genes are differentially regulated by both L14-8 and ezetimibe.

| **Table S1.** L14-8 regulated genes involved pathways. | | | | | |
| --- | --- | --- | --- | --- | --- |
| Term | Count | % | PValue | Fold Enrichment | FDR |
| Ferroptosis | 6 | 1.749 | 0.001 | 7.942 | 0.020 |
| Legionellosis | 6 | 1.749 | 0.003 | 5.957 | 0.043 |
| IL-17 signaling pathway | 10 | 2.915 | 0.000 | 5.852 | 0.003 |
| TNF signaling pathway | 12 | 3.499 | 0.000 | 5.606 | 0.001 |
| Pyrimidine metabolism | 5 | 1.458 | 0.020 | 4.793 | 0.138 |
| NF-kappa B signaling pathway | 9 | 2.624 | 0.001 | 4.765 | 0.017 |
| Lipid and atherosclerosis | 18 | 5.248 | 0.000 | 4.633 | 0.000 |
| Longevity regulating pathway - multiple species | 5 | 1.458 | 0.025 | 4.484 | 0.152 |
| Osteoclast differentiation | 11 | 3.207 | 0.000 | 4.307 | 0.008 |
| Protein processing in endoplasmic reticulum | 13 | 3.790 | 0.000 | 4.252 | 0.003 |
| Small cell lung cancer | 7 | 2.041 | 0.006 | 4.185 | 0.063 |
| Rheumatoid arthritis | 7 | 2.041 | 0.007 | 4.140 | 0.063 |
| Apoptosis | 10 | 2.915 | 0.001 | 4.088 | 0.019 |
| Fc epsilon RI signaling pathway | 5 | 1.458 | 0.035 | 4.029 | 0.198 |
| Autophagy - animal | 12 | 3.499 | 0.000 | 3.948 | 0.008 |
| Nucleotide metabolism | 6 | 1.749 | 0.018 | 3.925 | 0.129 |
| PPAR signaling pathway | 5 | 1.458 | 0.047 | 3.658 | 0.242 |
| T cell receptor signaling pathway | 8 | 2.332 | 0.006 | 3.646 | 0.063 |
| Non-alcoholic fatty liver disease | 10 | 2.915 | 0.002 | 3.541 | 0.035 |
| Breast cancer | 9 | 2.624 | 0.005 | 3.381 | 0.057 |
| Viral protein interaction with cytokine and cytokine receptor | 6 | 1.749 | 0.033 | 3.336 | 0.195 |
| Chagas disease | 6 | 1.749 | 0.037 | 3.239 | 0.206 |
| Estrogen signaling pathway | 8 | 2.332 | 0.012 | 3.200 | 0.098 |
| Systemic lupus erythematosus | 8 | 2.332 | 0.012 | 3.200 | 0.098 |
| Neutrophil extracellular trap formation | 11 | 3.207 | 0.002 | 3.185 | 0.035 |
| Mitophagy - animal | 6 | 1.749 | 0.040 | 3.177 | 0.210 |
| C-type lectin receptor signaling pathway | 6 | 1.749 | 0.040 | 3.177 | 0.210 |
| mTOR signaling pathway | 9 | 2.624 | 0.007 | 3.167 | 0.066 |
| Fluid shear stress and atherosclerosis | 8 | 2.332 | 0.013 | 3.154 | 0.102 |
| Alcoholism | 10 | 2.915 | 0.007 | 2.957 | 0.063 |
| Chemical carcinogenesis - reactive oxygen species | 12 | 3.499 | 0.002 | 2.952 | 0.035 |
| MAPK signaling pathway | 15 | 4.373 | 0.001 | 2.780 | 0.020 |
| Viral carcinogenesis | 10 | 2.915 | 0.011 | 2.712 | 0.097 |
| Prion disease | 13 | 3.790 | 0.004 | 2.600 | 0.051 |
| Transcriptional misregulation in cancer | 9 | 2.624 | 0.022 | 2.593 | 0.150 |
| Focal adhesion | 9 | 2.624 | 0.029 | 2.465 | 0.175 |
| Alzheimer disease | 16 | 4.665 | 0.004 | 2.275 | 0.051 |
| Parkinson disease | 11 | 3.207 | 0.023 | 2.257 | 0.150 |
| Pathways in cancer | 21 | 6.122 | 0.001 | 2.191 | 0.025 |
| Pathways of neurodegeneration - multiple diseases | 19 | 5.539 | 0.002 | 2.187 | 0.035 |

**Table S2.** Primers for RT-qPCR.

| **Primer name** | **5' to 3'** |
| --- | --- |
| qpreSAT1-F1 | ATGGGCGGGGAGGTAACTAA |
| qpreSAT1-R1 | AGCAAACAGACAGGAAGCGA |
| qpreSAT1-F2 | GGGCAATGCTGCTTCTTCTG |
| qpreSAT1-R2 | ACGTCAGGTTTACACGGCAG |
| qSAT1-F | AGATGGTTTTGGAGAGCACCC |
| qSAT1-R | GCCAATCCACGGGTCATAGG |
| qPLK1-F | TGACTCAACACGCCTCATCC |
| qPLK1-R | GCTCGCTCATGTAATTGCGG |
| qTP53-F | CCTCAGCATCTTATCCGAGTGG |
| qTP53-R | TGGATGGTGGTACAGTCAGAGC |
| qHMOX1-F | AGGGAATTCTCTTGGCTGGC |
| qHMOX1-R | CTTCGCCCCCTCTGAAGTTT |
| qALOXE3-F | CCAAGAACCCAAGGACCAGA |
| qALOXE3-R | GCTCCTGGATGTCCCTTGAG |
| qDPEP1-F | ACCAACATCCCCAAGCTGAG |
| qDPEP1-R | CCTGCACTGCTGGTGACATA |
| qNEAT1-F | GAGGGTGGGGAGTGAATGTG |
| qNEAT1-R | ACCACGGTCCATGAAGCATT |
| qATF3-F | ATTGTCCGGGCTCAGAATGG |
| qATF3-R | GGTTCTCTGCTGCTGGGATT |
| qPTGS2-F | AGGCTTCCATTGACCAGAGC |
| qPTGS2-R | TCCACAGCATCGATGTCACC |
| qFANCD2-F | TCAGCACACTGGCATTTAGC |
| qFANCD2-R | CATGATGCCAGCCATGGTCA |
| qSUV39H1-F | GAGTACCGTGTTGGTGAGGG |
| qSUV39H1-R | CTGGCCCTGGTCATTGTAGG |
| qAKR1C3-F | TCCGACCAGCCTTGGAAAAC |
| qAKR1C3-R | GTGAAAGTTCCTCACCTGGCT |
| qNUPR1-F | CCCAGCAATAGAGACGGGAC |
| qNUPR1-R | GGTAGCCCCTCAGAGACTCA |
| qRRM2-F | GGCTGGCTGTGACTTACCAT |
| qRRM2-R | AGCAGTGAGGCTGCATCTTT |
| qACTB-F | ACCGCGAGAAGATGACCCA |
| qACTB-R | GGATAGCACAGCCTGGATAGCAA |

Table S3. Primers for ChIP-qPCR.

| **Primer name** | **5' to 3'** |
| --- | --- |
| SAT1-ChIP-F1 | CAGTAGGGTTTCCGCCAAG |
| SAT1-ChIP-R1 | AACCCGGAGGACAAAAGTG |
| SAT1-ChIP-F2 | TCCTGAGTTTGCTTCCCACT |
| SAT1-ChIP-R2 | GGTGTGTCCCCCAGTAACAT |
| SAT1-ChIP-F3 | CACTGATTCTCAACTGCCAAA |
| SAT1-ChIP-R3 | CAGAAGCAGAGGAGGAAAAGG |
| SAT1-ChIP-F4 | CAAAAGACCACCCCTCACAT |
| SAT1-ChIP-R4 | CCTAGGGCAGGAAGGGTAAC |

Table S4. gRNA oligos for CRISPR-Cas13.

| **Oligos name** | **5' to 3'** |
| --- | --- |
| TP53-gF1 | AAACGATTCTCTTCCTCTGTGCGCCG |
| TP53-gR1 | AAAACGGCGCACAGAGGAAGAGAATC |
| TP53-gF2 | AAACGAAGTAGTTTCCATAGGTCTGA |
| TP53-gR2 | AAAATCAGACCTATGGAAACTACTTC |
| TP53-gF3 | AAACTTCTGGGAGCTTCATCTGGACC |
| TP53-gR3 | AAAAGGTCCAGATGAAGCTCCCAGAA |
| TP53-gF4 | AAACTTACATCTCCCAAACATCCCTC |
| TP53-gR4 | AAAAGAGGGATGTTTGGGAGATGTAA |
| SAT1-gF1 | AAACAATAACTTGCCAATCCACGGGT |
| SAT1-gR1 | AAAAACCCGTGGATTGGCAAGTTATT |
| SAT1-gF2 | AAACATTTCTGATCCTATGCCAAAGC |
| SAT1-gR2 | AAAAGCTTTGGCATAGGATCAGAAAT |
| SAT1-gF3 | AAACTTAGCAAGTACTCCTTGTCGAT |
| SAT1-gR3 | AAAAATCGACAAGGAGTACTTGCTAA |
| SAT1-gF4 | AAACCTATAATCACTCATCACGAAGA |
| SAT1-gR4 | AAAATCTTCGTGATGAGTGATTATAG |

Table S5. Predicted targets of L14-5.

| **Target** | **Common name** | **Uniprot ID** | **ChEMBL ID** |
| --- | --- | --- | --- |
| Cannabinoid receptor 1 | CNR1 | P21554 | CHEMBL218 |
| Type-1 angiotensin II receptor | AGTR1 | P30556 | CHEMBL227 |
| Hexokinase type IV | GCK | P35557 | CHEMBL3820 |
| Phosphodiesterase 10A | PDE10A | Q9Y233 | CHEMBL4409 |
| Scavenger receptor class B member 1 | SCARB1 | Q8WTV0 | CHEMBL1914272 |
| Serine/threonine-protein kinase PLK1 | PLK1 | P53350 | CHEMBL3024 |
| Cyclooxygenase-2 | PTGS2 | P35354 | CHEMBL230 |
| Phosphodiesterase 4D | PDE4D | Q08499 | CHEMBL288 |
| Elongation of very long chain fatty acids protein 6 | ELOVL6 | Q9H5J4 | CHEMBL5704 |
| Vascular endothelial growth factor receptor 2 | KDR | P35968 | CHEMBL279 |
| G-protein coupled receptor kinase 2 | GRK2 | P25098 | CHEMBL4079 |
| Complement factor D | CFD | P00746 | CHEMBL2176771 |
| TGF-beta receptor type I | TGFBR1 | P36897 | CHEMBL4439 |
| C5a anaphylatoxin chemotactic receptor | C5AR1 | P21730 | CHEMBL2373 |
| Kir3.1/Kir3.4 | KCNJ5 | P48544 | CHEMBL3038488 |
| Voltage-gated potassium channel subunit Kv1.5 | KCNA5 | P22460 | CHEMBL4306 |
| Serine/threonine-protein kinase PLK3 | PLK3 | Q9H4B4 | CHEMBL4897 |
| Serine/threonine-protein kinase PLK2 | PLK2 | Q9NYY3 | CHEMBL5938 |
| Macrophage colony stimulating factor receptor | CSF1R | P07333 | CHEMBL1844 |
| Tyrosine-protein kinase JAK3 | JAK3 | P52333 | CHEMBL2148 |
| Discoidin domain-containing receptor 2 | DDR2 | Q16832 | CHEMBL5122 |
| Cathepsin (V and K) | CTSV | O60911 | CHEMBL3272 |
| Angiotensin II receptor | AGTR2 | P50052 | CHEMBL4607 |
| Poly [ADP-ribose] polymerase 10 | PARP10 | Q53GL7 | CHEMBL2429708 |
| Urokinase plasminogen activator surface receptor | PLAUR | Q03405 | CHEMBL4883 |
| Platelet-derived growth factor receptor beta | PDGFRB | P09619 | CHEMBL1913 |
| Isocitrate dehydrogenase [NADP] cytoplasmic | IDH1 | O75874 | CHEMBL2007625 |
| 6-phosphofructo-2-kinase/fructose-2,6-bisphosphatase 3 | PFKFB3 | Q16875 | CHEMBL2331053 |
| Tyrosine-protein kinase SRC | SRC | P12931 | CHEMBL267 |
| Kininogen-1 | KNG1 | P01042 | CHEMBL3638337 |
| Tyrosine-protein kinase SYK | SYK | P43405 | CHEMBL2599 |
| Serine/threonine-protein kinase B-raf | BRAF | P15056 | CHEMBL5145 |
| Serine/threonine-protein kinase TNNI3K | TNNI3K | Q59H18 | CHEMBL5260 |
| Purinergic receptor P2Y12 | P2RY12 | Q9H244 | CHEMBL2001 |
| Proteinase-activated receptor 1 | F2R | P25116 | CHEMBL3974 |
| 5-lipoxygenase activating protein | ALOX5AP | P20292 | CHEMBL4550 |
| Coagulation factor IX | F9 | P00740 | CHEMBL2016 |
| Probable G-protein coupled receptor 142 | GPR142 | Q7Z601 | CHEMBL2069161 |
| Gamma-secretase | PSEN2 | P49810 | CHEMBL2094135 |
| Adenosine A1 receptor | ADORA1 | P30542 | CHEMBL226 |
| Adenosine A2a receptor | ADORA2A | P29274 | CHEMBL251 |
| Adenosine A2b receptor | ADORA2B | P29275 | CHEMBL255 |
| Phosphodiesterase 4B | PDE4B | Q07343 | CHEMBL275 |
| Phosphodiesterase 4C | PDE4C | Q08493 | CHEMBL291 |
| PI3-kinase p110-delta subunit | PIK3CD | O00329 | CHEMBL3130 |
| Metabotropic glutamate receptor 5 | GRM5 | P41594 | CHEMBL3227 |
| Calcitonin gene-related peptide type 1 receptor | CALCRL | Q16602 | CHEMBL3798 |
| PI3-kinase p110-alpha subunit | PIK3CA | P42336 | CHEMBL4005 |
| CDC7/DBF4 (Cell division cycle 7-related protein kinase/Activator of S phase kinase) | CDC7 | O00311 | CHEMBL5443 |
| Thromboxane-A synthase | TBXAS1 | P24557 | CHEMBL1835 |

**Reference**

1. Q. He *et al.*, Ezetimibe inhibits triple-negative breast cancer proliferation and promotes cell cycle arrest by targeting the PDGFR/AKT pathway. *Heliyon* **9**, e21343 (2023).
